# Inferring Population Histories for Ancient Genomes Using Genome-Wide Genealogies

**DOI:** 10.1093/molbev/msab174

**Published:** 2021-06-15

**Authors:** Leo Speidel, Lara Cassidy, Robert W Davies, Garrett Hellenthal, Pontus Skoglund, Simon R Myers

**Affiliations:** 1 Francis Crick Institute, London, United Kingdom; 2 Genetics Institute, University College London, London, United Kingdom; 3 Smurfit Institute of Genetics, Trinity College Dublin, Dublin, Republic of Ireland; 4 Department of Statistics, University of Oxford, Oxford, United Kingdom; 5 Wellcome Centre for Human Genetics, University of Oxford, Oxford, United Kingdom

**Keywords:** population genetics, ancient genomes, mutation rate evolution, genealogies

## Abstract

Ancient genomes anchor genealogies in directly observed historical genetic variation and contextualize ancestral lineages with archaeological insights into their geography and cultural associations. However, the majority of ancient genomes are of lower coverage and cannot be directly built into genealogies. Here, we present a fast and scalable method, *Colate*, the first approach for inferring ancestral relationships through time between low-coverage genomes without requiring phasing or imputation. Our approach leverages sharing patterns of mutations dated using a genealogy to infer coalescence rates. For deeply sequenced ancient genomes, we additionally introduce an extension of the *Relate* algorithm for joint inference of genealogies incorporating such genomes. Application to 278 present-day and 430 ancient DNA samples of >0.5x mean coverage allows us to identify dynamic population structure and directional gene flow between early farmer and European hunter-gatherer groups. We further show that the previously reported, but still unexplained, increase in the TCC/TTC mutation rate, which is strongest in West Eurasia today, was already present at similar strength and widespread in the Late Glacial Period ~10k−15k years ago, but is not observed in samples >30k years old. It is strongest in Neolithic farmers, and highly correlated with recent coalescence rates between other genomes and a 10,000-year-old Anatolian hunter-gatherer. This suggests gene-flow among ancient peoples postdating the last glacial maximum as widespread and localizes the driver of this mutational signal in both time and geography in that region. Our approach should be widely applicable in future for addressing other evolutionary questions, and in other species.

## Introduction

Genetic variation is shaped through evolutionary processes acting on our genomes over hundreds of millennia, including past migrations, isolation by distance, mutation, or recombination rate changes, and natural selection. Such events are reflected in the genealogical trees that relate individuals back in time. Although these are unobserved, recent advances have made their reconstruction from genetic variation data increasingly feasible, with the most scalable methods now able to build trees for many thousands of individuals (Kelleher et al. 2019; Speidel et al. 2019). This has enabled powerful inferences of our genetic past (Rasmussen et al. 2014; Kelleher et al. 2019; Speidel et al. 2019).

Ancient genomes provide a direct snapshot of historical genetic variation, and so add substantial information compared with genealogies built only from modern-day samples. We introduce an extension to the *Relate* algorithm to enable the incorporation of samples of variable ages. We use this approach to reconstruct joint genealogies of the Simon’s Genome Diversity Project (SGDP) data set (Mallick et al. 2016) and 14 previously published high-coverage ancient humans covering diverse ancestries and sampled across the last 45k years (Fu et al. 2014; Lazaridis et al. 2014; Gallego-Llorente et al. 2015; Jones et al. 2015; Broushaki et al. 2016; Sikora et al. 2017; de Barros Damgaard et al. 2018; Günther et al. 2018; Sikora et al. 2019; Cassidy et al. 2020). These genealogies capture the shared population histories of present-day and ancient humans. In particular, they allow identification of inbreeding, directional migration, and estimation of coalescence rates between individuals, analysis of the age and spread of individual mutations, and in future might be used to infer natural selection (Speidel et al. 2019). A similar approach could also be applied to other species.

The joint inference of genealogies for ancients and moderns currently requires accurate diploid genotypes, and thus excludes the majority of ancient human genomes, because these have lower sequencing coverage. One central set of questions for such samples involve estimation of their joint genetic history: Their historical relationships with one another through time, reflected in their varying coalescence rates through time. These coalescence rates can be estimated using a number of methods (Gutenkunst et al. 2009; Li and Durbin 2011; Schiffels and Durbin 2014; Terhorst et al. 2017; Kamm et al. 2020), as well as our updated *Relate* approach, but to date none of these have been designed to work for low-coverage genomes. We have therefore developed a fast and scalable method, *Colate*, for inferring coalescence rates between low-coverage genomes without requiring phasing or imputation. *Colate* leverages mutational ages obtained from a *Relate*-inferred genealogy to construct a likelihood based on the changing pattern of sharing of mutations through time, which we maximize using an expectation-maximization (EM) algorithm. The method can calculate coalescence rates between any number of samples. Running *Colate* involves two steps: First, a data parsing and preprocessing step whose complexity is linear in sample size and genome length and secondly a constant time (∼5 s, see Materials and Methods) analysis step to run the EM algorithm.

We applied *Colate* to 430 genomes of >0.5x coverage spanning the late Paleolithic, Mesolithic, Neolithic, and more recent epochs across many regions outside Africa (supplementary table 1, Supplementary Material online). Among other findings, we readily identify genetic clusters corresponding to hunter-gatherers (HGs), Early farmers, and the Late Neolithic-Bronze age transition in Europe, and map out the coalescence rates of modern humans worldwide with these ancient samples. We show that these indicate localized structure which converges back in time, and characterize dramatic population replacements in Ireland within the space of 3,000 years. A strength of the *Relate* and *Colate* approaches is that they extrapolate relationships of individuals to the past where data are comparatively sparse. We find evidence of directional gene flow between European HG groups across Europe predating the Neolithic, which is more widespread than previously identified.

Finally, we leverage our *Relate*-inferred genealogies and *Colate-*inferred coalescence rates to quantify the previously reported but unexplained elevation in TCC to TTC mutation rate (Harris 2015) in all SGDP individuals and 161 ancient individuals of >2x mean coverage, providing a finer-scale geographic and temporal mapping of this signal than previously available. We show that the signal shows a remarkable 96% correlation with coalescence rates with an early Anatolian farmer from the prepottery Neolithic (Kılınç et al. 2016). Although absent in samples from >34,000 years before present (YBP), it was already widespread among HGs in Late Glacial West Eurasia, and shows no increase in strength over the last 10,000 years, suggesting that the driver of this mutational signature was extinct by the Holocene. This strong localization of the signal in both time and space suggests either a genetic cause, or a somehow tightly focused environmental cause. Moreover, we hypothesize that these excess TCC/TTC mutations spread via gene flow through ancestors of ancient Anatolia into HG groups across Western Eurasia before the expansion of farming, perhaps associated with a link between the Near East and Late Upper Paleolithic Europe that started with the Bølling–Allerød interstadial warming period (Fu et al. 2016).

## New Approaches

### Extending *Relate* to Work with Noncontemporary Samples

We extend our previously developed method, *Relate*, for inference of genealogical trees genome-wide for large sample sizes (Speidel et al. 2019) to work with ancient genomes (supplementary information, Supplementary Material online). A key aspect of noncontemporary samples is that, when these samples have known ages, these impose hard constraints on the times of coalescence events. Our updated tree builder restricts which lineages can coalesce by assigning a preliminary date to each coalescence event and only allows coalescences of noncontemporary samples with lineages that predate its age. Once tree topologies are inferred, branch lengths are sampled using a Markov-Chain Monte Carlo approach, with proposal distributions that allow for noncontemporary samples. As in our previous versions (Speidel et al. 2019), we sample branch lengths from a posterior distribution that fixes tree topology and combines the likelihood of observing a certain number of mutations on a branch and a coalescent prior with piecewise-constant effective population sizes through time.

### Inferring Coalescence Rates for Low-Coverage Genomes Using *Colate*

**Fig. 1.**
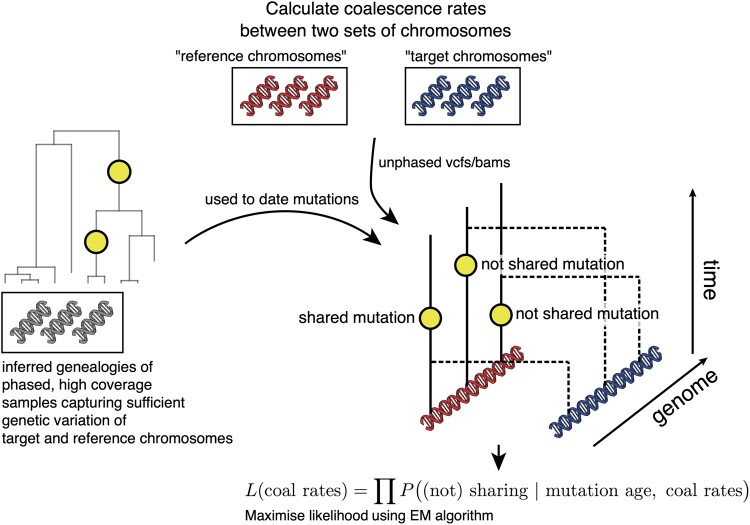
*Colate* calculates coalescence rates between two sets of chromosomes, labeled target and reference (main text). The method proceeds by recording for each mutation carried by a reference chromosome, whether it is shared in the target chromosomes. This information is summarized in a likelihood, constructed by multiplying over SNPs, such that no phase information is required. Whenever more than one chromosome is available at any given site, we multiply across chromosomes. The likelihood is maximized using an EM algorithm.

*Colate* calculates coalescence rates between a set of “target” and a set of “reference” chromosomes by leveraging mutations dated using an inferred genealogy. This genealogy may (or may not) have overlapping samples with the target and reference chromosome sets (fig. 1; see Materials and Methods; supplementary information, Supplementary Material online). Both the target and reference chromosomes may be specified as BCF files containing unphased genotypes, or as BAM files containing reference-aligned reads. The latter is particularly useful for low-coverage sequencing data, where accurate genotype calling is not possible. For ancient genomes, we specify a sample date. In practise, we often specify two different individuals as the target and reference, and obtain the coalescence rates between this pair, although it is also possible to group samples.

The *Colate* likelihood uses as input data whether each mutation carried by a reference chromosome is shared, or not shared, with a target chromosome. Sharing indicates that coalescence between the two chromosomes happened more recently than the age of this mutation, whereas nonsharing indicates that coalescence happened further in the past, assuming each mutation occurs only once (the infinite-sites model), and so an exact likelihood can be calculated, given coalescence rates between the individuals from whom these chromosomes are taken (see Materials and Methods). We multiply this likelihood across sites and therefore do not require genomes to be phased; in low-coverage data, we additionally multiply across pairs of reads. This likelihood is then maximized using an EM algorithm (see Materials and Methods, supplementary information, Supplementary Material online). Our implementation reduces computation time by using a discrete time grid to record sharing and nonsharing of mutations through time, reducing the computation time of the EM algorithm. As a result, computation time is independent of both sample size and genome lengths once the data are preprocessed, and typically on the order of 5 s (∼40 s including parsing the data, supplementary fig. 1, Supplementary Material online).

**Fig. 2.**
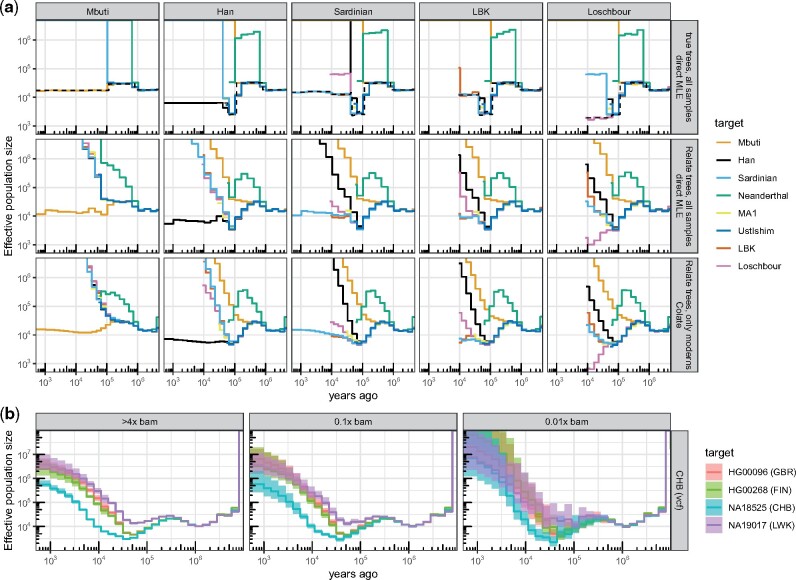
(*a*) Simulation emulating real human groups, including three modern human groups (Mbuti, Han, and Sardinian) with 100 diploid sequences each, and five diploid ancient genomes. We calculated coalescence rates between groups using true genealogical trees of all samples (true trees; direct MLE), inferred *Relate* trees of all samples (*Relate* trees; direct MLE), and *Colate*, where the genealogy used to date mutations included all modern human groups but not the ancient samples. For the direct MLEs, coalescence rates are symmetric with respect to target and reference group assignment; for *Colate*, each panel corresponds to a fixed reference group, with different colored lines showing different target groups. Five reference groups are shown here, see supplementary figure 3, Supplementary Material online, for remaining groups. Dashed lines show true within-group population sizes. (*b*) *Colate*-inferred coalescence rates between four 1000 Genomes Project samples (HG0096, HG00268, NA18525, NA19017) and the remaining 1000 Genomes samples of Han Chinese in Beijing (CHB) (see supplementary fig. 3, Supplementary Material online, for rates to YRI and CEU). The target samples are given as reference-aligned read data downsampled to 4x, 0.1x, and 0.01x mean coverage. Confidence intervals are constructed using 100 block bootstrap iterations with a block size of 20 Mb.

We observe high accuracy of *Colate* and *Relate*-inferred coalescence rates using the stdpopsim package (Adrion et al. 2020), on simulated data following a zigzag demographic history (supplementary fig. 2, Supplementary Material online) as well as a multipopulation model of ancient Eurasia, which was fitted using real human genomes (Kamm et al. 2020) (fig. 2*a*;supplementary fig. 3, Supplementary Material online) (see Materials and Methods; Speidel et al. (2019) for comparison of *Relate* to other methods). Relate coalescence rates encourigingly show that ancient genomes are coalesced into the genealogies at expected rates and correct order on average. We further evaluated *Colate*’s performance on low-coverage sequencing data by downsampling high-coverage genomes of the 1000 Genomes Project (1000 Genomes Project Consortium 2015). Although uncertainty increases as coverage decreases, *Colate* recovers meaningful coalescence rate estimates even between a sequence of 0.01x mean coverage and high-coverage sequences specified as a BCF (fig. 2*b*), or between two low-coverage sequences of 0.1x mean coverage (supplementary fig. 4, Supplementary Material online).

## Results

### *Relate* and *Colate* Applied to 278 SGDP Moderns and 430 Ancients

We inferred joint genealogies of 278 modern-day individuals of the SGDP and 14 previously published high coverage genomes of ancient individuals of >8x mean coverage, which we collectively rephase using Shapeit4 (Delaneau et al. 2019) and the 1000 Genomes Project reference panel (see Materials and Methods). Tree topologies were constructed using all mutations except CpG dinucleotides, but branch length inference used transversions only, so as to avoid confounding due to deamination errors in the ancient genome sequences (see Materials and Methods). Furthermore, we estimate pairwise-coalescence rates for 430 ancient individuals of >0.5x mean sequencing coverage using *Colate* (supplementary table 1, Supplementary Material online). For *Colate*, we use a *Relate*-inferred genealogy of the SGDP samples to date mutations, sampling one haplotype from each individual to remove the effects of recent inbreeding and restrict our analysis to transversions (see Materials and Methods).

### PCA on *Colate*-Inferred Coalescence Rates Captures Dynamic Population Structure

**Fig. 3.**
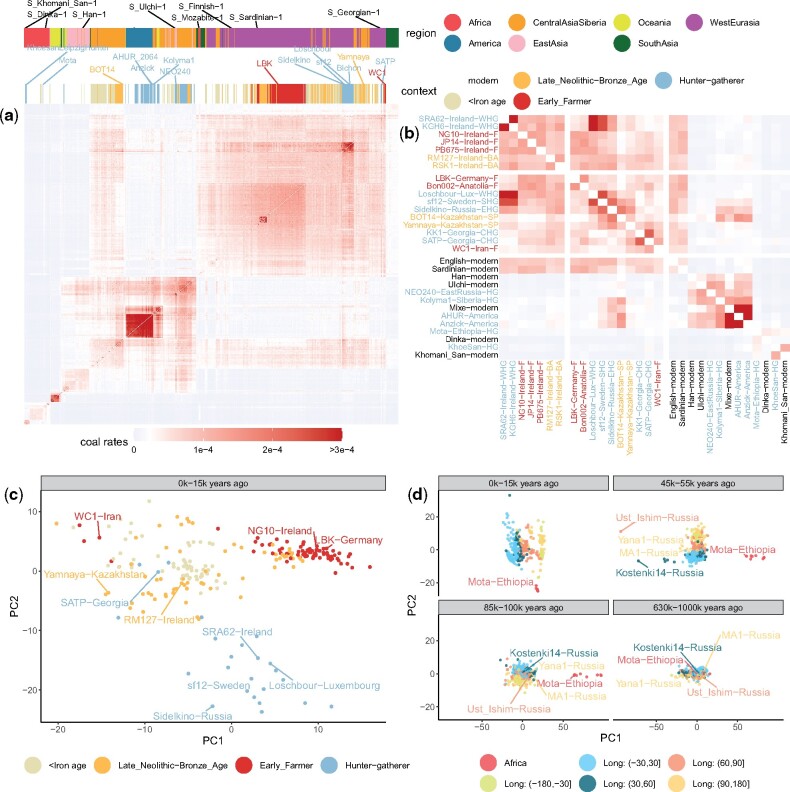
(*a*) Matrix of *Colate*-inferred pairwise coalescence rates for all modern SGDP individuals and ancient individuals in the most recent epoch 0 − 15,000 YBP. (*b*) Highlighted subset of samples from (*a*). Sample names are colored by context. Abbreviations in sample names are WHG, Western hunter-gatherer; SHG, Scandinavian hunter-gatherer; EHG, Eastern hunter-gatherer; CHG, Caucasus hunter-gatherer; F, farmer; BA, Bronze Age, SP, Steppe Pastoralists. (*c*) Principal component analysis (PCA) on pairwise coalescence rates of ancient individuals in West Eurasia in epoch 0–15,000 YBP, colored by context. (*d*) PCA on pairwise coalescence rates for four epochs, colored by Longitude outside Africa. In all PCAs, we standardized columns in each matrix of coalescence rates and applied the R function prcomp to calculate PCs.

*Colate*-inferred coalescence rates demonstrate intricate relationships that vary geographically and through time and manifest vast migrations and, in places, repeated population replacements (fig. 3*a* and *b*). In the recent past (0 − 15 KY), populations are separated based on both geography and sample age (fig. 3*a* and *b*): There are extremely low coalescence rates between continental regions (excepting W. Eurasia and Central Asia, which show patterns indicating migration). Taking samples from Ireland as one example (fig. 3*b*), previous work has indicated repeated partial or complete population replacements, first of Mesolithic HGs by Neolithic farmers, and then in the Bronze age by migrants related to people from the Western steppe (Cassidy et al. 2016). Using *Colate*, the earliest Irish Mesolithic samples have highest coalescence rates with, and similar relatedness to other groups as, West European HGs (e.g., Loschbour). Neolithic Irish samples show much lower affinity to these HGs, but are closely similar to other European farmers (e.g., LBK, an early farmer from Germany). Bronze age Irish samples again show more similarity to HGs, but now *Eastern* European HGs (and other Eastern European groups), and in this and other respects they resemble the Yamnaya, a possible source group (fig. 3*b*); however, they retain some farmer-like haplotypes not present in the Yamnaya sample. Comparing across the whole data set, we observe that Irish ancient genomes are closest to other Irish ancients from within the same time period (supplementary figs. 5 and 6, Supplementary Material online). This implies that finer scale, regional stratification existed within the HGs, Neolithic farmers, and Bronze age samples, but there is no clear evidence of continuity across periods, suggesting this arose independently repeatedly. We also identify clear substructure among European HGs, consistent with previous findings (Lazaridis et al. 2014) and pairwise F2 statistics (supplementary fig. 7, Supplementary Material online); this structure corresponds to a divide of Western, Eastern, Scandinavian, and Caucasus HGs among our samples in Europe.

One approach to visualize the diverse signals in these data is to adapt the widely used PCA approach, but now using coalescence rates within particular epochs (fig. 3*c* and *d* shows the first two PCs for selected epochs). Structure is not seen in the deep past (>630k YBP) but in distinct epochs we observe separation first of African (e.g., Mota) and non-African individuals, and by 45 − 55k YBP, a separation between West and East Eurasians, as well as a stronger split with Ust’-Ishim (Fu et al. 2014), a 45k-year-old Siberian individual who also appears slightly closer to East Eurasians compared with later European samples, such as Kostenki14 (Seguin-Orlando et al. 2014) and Sunghir3 (Sikora et al. 2017), who are closer to West Eurasians. In the most recent epoch (0 − 15k YBP), our PCA mirrors geography globally (Novembre et al. 2008), but reflects different ancestries more strongly within smaller regions; for instance, we detect three clusters, corresponding to Mesolithic HGs, Neolithic farmers, and Bronze/Iron age individuals in Europe (fig. 3*c*). The Bronze age cluster falls closer to Steppe Pastoralists from the Pontic-Caspian Steppe (e.g., Yamnaya), consistent with previously reported gene flow from this region into Bronze age Europe (Allentoft et al. 2015; Haak et al. 2015). Overall, these inferences seem in strong agreement, across time, and space, with previous specific analyses of these samples.

### Relationship of European HG Groups to Neolithic Farmers

**Fig. 4.**
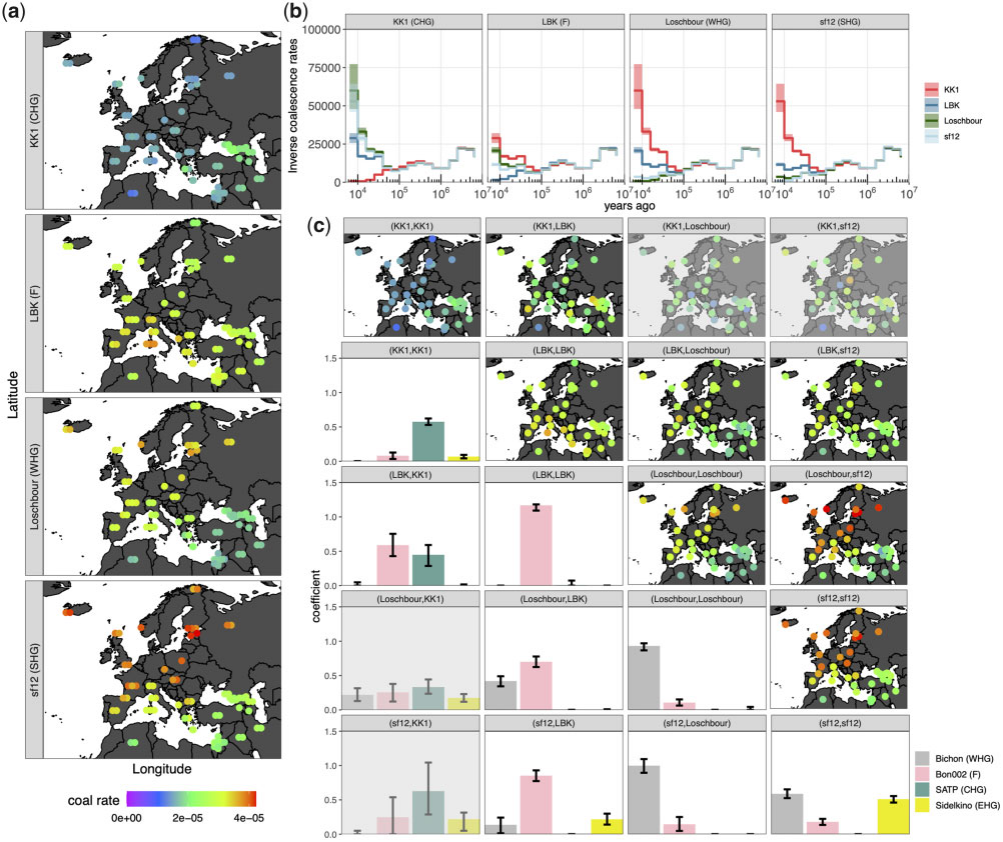
(*a*) Map showing *Relate*-inferred coalescence rates of a 9,700-year-old Caucasus HG (KK1), 7,200-year-old early European farmer (LBK), a nearly 8,000-year-old Western hunter-gatherer (Loschbour), and a 9,000-year-old Scandinavian HG to SGDP modern individuals. The coalescence rates shown in the map correspond to the epoch 16k−25k YBP. (*b*) *Relate*-inferred inverse coalescence rates (effective population sizes) for KK1, LBK, Loschbour, and sf12 to themselves and each of the other four individuals. (*c*) Maps in top diagonal show *Relate*-inferred coalescence rates of lineages with descendants shown by facet labels to SGDP moderns in same epoch as in (*a*). Bottom diagonal shows nonlinear least squares coefficients obtained by fitting coalescence rates of lineages with descendants given by facet labels to SGDP moderns as a mixture of *Colate*-inferred coalescence rates of Bichon (Western HG), Bon002 (Anatolian), SATP (Caucasus HG), Sidelkino (Eastern HG) with SGDP moderns (see Materials and Methods). Panels involving KK1 and Loschbour or sf12 are partially grayed out, as there is little recent gene-flow between these groups. Confidence intervals show 2.5 and 97.5 percentiles obtained from 1,000 bootstrap samples.

Although there is strong evidence for Anatolian farmers partially replacing HG ancestry across Europe in the Neolithic (Haak et al. 2010), the deeper relationship of ancestors of these Anatolian farmers to European HGs in the Late Upper Paleolithic is not fully understood. We therefore assess these deep relationships between early European farmers, Western, Scandinavian, and Caucasus HGs built into our *Relate* genealogies. These groups show distinct footprints in present-day Europeans, consistent with previous findings (fig. 4). We observe a South-North cline, with the highest farmer-like ancestry observed in Sardinians (fig. 3*b*), whereas Western and Scandinavian HG-like ancestry is highest in northern European groups and Caucasus HG-like ancestry is concentrated around present-day Georgia (Lazaridis et al. 2014; Skoglund et al. 2014; Jones et al. 2015).

Caucasus HGs have previously been modeled as forming a clade with early farmers that is deeply diverged from Western and Scandinavian HGs (∼46k YBP) (Jones et al. 2015). Our pairwise coalescence rates among samples confirm that Western and Scandinavian HGs form a clade relative to Caucasus HGs (KK1), with a consistent split time and almost no recent coalescences observed between these groups (fig. 4*b*), however patterns observed for early farmers (LBK) imply a nontree-like group relationship involving migration:

Caucasus HGs show greater affinity to Neolithic farmers than to Western or Scandinavian HGs in recent epochs, but this is not reciprocated by early farmers who have higher coalescence rates to Western and Scandinavian HGs than to Caucasus HGs (fig. 4*b*). This could reflect observations in recent studies that found that the major ancestral component of Western HGs only became widespread in Northern and Western Europe after 14k YBP and harbors an increased affinity to Anatolian and Caucasus populations, relative to earlier European HGs (Fu et al. 2016), suggesting an expansion of peoples from Southeast Europe or the Near East following the last glacial maximum (LGM) but predating the European Neolithic.

To test for evidence of such migration between ancestors of early farmers and other European HGs, we examine lineages that are formed recently (<50k YBP) through a coalescence of individuals from each group. If this coalescence happened more recently than the split time of groups A and B, these lineages are expected to represent migrants from one population to another. If recent migration is purely directional from group A into group B, such lineages will always come from group A in the past and will behave like a typical group A lineage back in time. We therefore evaluate whether these recently coalesced lineages are more similar to a typical lineage ancestral to group A or group B by comparing their coalescence rates to other individuals, as this should distinguish their affiliation (group A lineages can be characterized as coalescing rapidly with some individuals, while group B lineages coalescece more slowly with the same individuals, see Materials and Methods). To gain power, we calculate the coalescence rates to each non-African SGDP modern sample and fit these using non-negative least squares against *Colate*-inferred coalescence rates (also calculated against SGDP samples) of four individuals representing independent samples from similar, but older groups: Ancient Anatolia (Bon002) (Kılınç et al. 2016), Western HGs (Bichon) (Jones et al. 2015), Eastern HGs (Sidelkino) (de Barros Damgaard et al. 2018), and Caucasus HGs (SATP) (Jones et al. 2015) (fig. 4*c*, see Materials and Methods). This will fit these recently coalesced lineages as a mixture of four potential surrogate source populations. We rescaled *Colate* coalescence rates according to supplementary figure 9, Supplementary Material online to match overall levels of coalescence rates between *Colate* and *Relate*.

Encouragingly, we find that lineages ancestral to the two haplotypes of the same individual (not indicating migration) are well captured by one respective ancestry in our regression in three cases and suggests these are reasonable surrogates. The exception is the Scandinavian HG (sf12) who we fit as an approximately equal mixture of Eastern and Western HGs, as previously reported (Günther et al. 2018). The highest recent coalescence rates across groups are between the Western and Scandinavian HG: Recently coalesced lineages between these samples appear very similar to Western HGs (fig. 4*c*), indicating strong directionality of gene-flow, from Western HGs into Scandinavia. In contrast, gene-flow between Western HGs (Loschbour) and early farmers (LBK) appears strongly bidirectional in our analysis, as do lineages ancestral to LBK and Scandinavian or Caucasus HGs, therefore suggesting widespread migration between ancestors of these groups predating the European Neolithic.

### Effective Population Sizes Increased from Mesolithic Europe to the Present

**Fig. 5:**
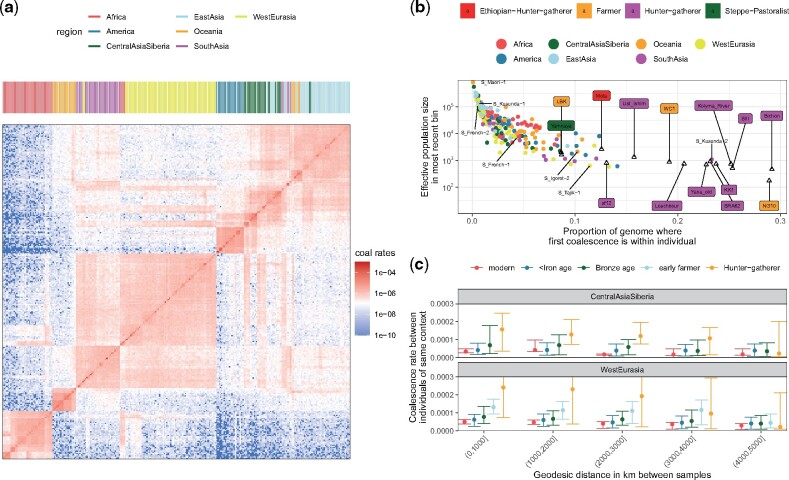
(a) *Relate*-inferred coalescence rates between SGDP individuals in the most recent epoch (0–1,000 YBP). (*b*) Within individual effective population sizes in the most recent epoch vs. the proportion of the genome where the first coalescence occurs within the individual. All coalescence rates are calculated using *Relate* trees. (*c*) *Colate*-inferred coalescence rates in the most recent epoch (<15k YBP) for pairs of samples grouped by geographic distance and time period. Circles indicate median, error bars show the 2.5% and 97.5% percentiles, respectively.

Effective population sizes calculated within an individual quantify diversity and relatedness of parental genomes. By focusing on the very recent past (<1000 years), we observe a broad spectrum of recent within-individual effective population sizes in SGDP individuals ranging from a few thousand to hundreds of thousands not limited to particular geographical groups (fig. 5*a*;supplementary fig. 8, Supplementary Material online) and correlating well between *Relate* and *Colate* (supplementary fig. 9, Supplementary Material online). Haplotypes of individuals with small recent effective population sizes coalesce with each other before coalescing with any other sample for larger proportions of the genome (fig. 5*b*), indicative of longer runs of homozygosity (ROH) in these individuals (supplementary fig. 10, Supplementary Material online). Although global patterns are comparable to previously reported heterozygosity estimates (Mallick et al. 2016), the differences among particular individuals are more pronounced in our analysis, which focuses on very recent time.

Small recent effective population sizes are also observed in the high coverage ancient genomes and are most pronounced in European Mesolithic HGs, who also tend to coalesce with themselves for larger proportions of the genome. However, this may at least in part be driven by increased divergence from other samples analyzed, in addition to ROH (fig. 5*b*). Despite the larger effective population sizes between unrelated chromosomes of Neolithic contexts, relatively, to those of HG contexts, the smallest recent effective population size is observed for the NG10 individual, a 5,200-year-old Neolithic individual buried in a Megalithic tomb in Ireland. This individual was previously identified to be the son of first-degree relatives (Cassidy et al. 2020).

We next compared coalescence rates across individuals at increasing geographic distances within Europe, and within Central Asia, in each time period, including only modern individuals within 500 km of an ancient sample (fig. 5*c*). At shorter distances we observe a clear trend for smaller coalescence rates (larger effective population sizes) toward the present, suggesting strongly increasing local population sizes. At larger distances, the relationship is nonmonotonic, with coalescence rates not decreasing consistently, implying a trend of increasing migration, countering the larger population sizes. Finally, we see a trend of decreasing similarity with distance, implying local population structure at all times, with the interesting exception of samples more recent than the beginning of the Iron age (yet not modern) in Europe. More widespread sampling is needed to understand this pattern, although this period does overlap, for example, increased mobility during the Roman Empire and the following “migration age” in Europe characterized by widespread movements of peoples (Martiniano et al. 2016).

### Elevation in TCC to TTC Mutation Rate Is Present in Mesolithic HGs and Neolithic Farmers

The triplet TCC has seen a remarkable increase in mutation rates toward TTC in humans, first identified by Harris (2015). This signature has no known cause to date and appears strongest in Europeans and weaker in South Asians. It was previously estimated to have started around 15k−20k YBP, and its driver is most likely absent in present-day individuals (Harris and Pritchard 2017; Speidel et al. 2019), although there is considerable uncertainty about this estimate—for example, a recent study dates the onset to up to ∼80k YBP depending on the demographic history used (DeWitt et al. 2021). One study previously quantified the signal in an early farmer (LBK) and Western HG (Loschbour), suggesting that both carried the signal, whereas the signal was missing in Ust’-Ishim, Neanderthals, and Denisovans (Mathieson and Reich 2017).

**Fig. 6:**
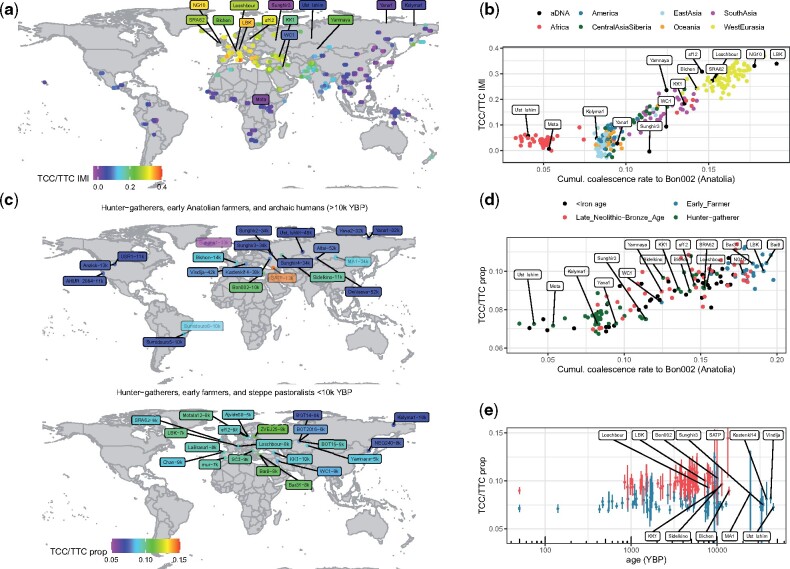
(a) Map showing the strength of the TCC/TTC mutation rate signature, quantified by calculating the “IMI” of the TCC/TTC mutation rate (see Materials and Methods). Circles correspond to present-day individuals in the SGDP data, ancient individuals are labeled. (*b*) TCC/TTC IMI plotted against the *Colate*-inferred coalescence rates to Bon002, a 10k-year-old individual from Anatolia, integrated between 14k and 50k YBP. Circles correspond to SGDP samples, labels to ancients. (*c*) Map showing the TCC/TTC mutation rate signature in lower coverage ancients, quantified as the proportion of sites that are TCC/TTC relative to other C/T transitions excluding those in CpG contexts (see Materials and Methods). Top shows a subset of samples >10k years old, bottom shows samples <10k years old (see supplementary fig. 13, Supplementary Material online, for further samples). Samples of <2x mean coverage are shown with increased transparency. Number following sample ID shows sample age. (*d*) Proportion of TCC/TTC sites plotted against coalescence rates to Bon002, integrated between 14k and 50k YBP. All points correspond to ancients, color indicates their age. (*e*) Proportion of TCC/TTC sites plotted against sample age. Confidence intervals are obtained using a block bootstrap. Samples are colored using a k-means clustering (k = 2). In (*c*, *d*, *e*), samples are >2x mean coverage, except for those >10k years old where we included samples >1x mean coverage.

We first inferred the rate through time at which TCC mutates toward TTC in every individual built into our genealogy of moderns and ancients, after excluding singletons, and then quantified signal strength by calculating the “integrated mutation intensity” (IMI) which quantifies the area under the mutation rate curve (see Materials and Methods). Among SGDP individuals, the quantified signal varies and is strongest in Southern Europeans such as Sardinians, who are known to have an increased affinity to early Neolithic farmers (fig. 6*a*, supplementary fig. 11, Supplementary Material online). Among the high-coverage ancients built into our *Relate* genealogies, we observe the signature in Mesolithic HGs, as well as in Neolithic and Bronze age samples, including the Yamnaya (fig. 6*a*), but infer it to be weaker in HGs and strongest in Neolithic farmers. The signal is absent in an Ethiopian HG, as expected, as well as in both the 45,000 year old Ust’-Ishim sample and the 34,000 year-old Sunghir3 sample (fig. 6*a*).

To quantify the signal in individuals of lower coverage, we calculate the proportion of TCC/TTC mutations relative to C/T transitions in each individual, restricting to mutations ascertained in SGDP samples, of at least 4x coverage in the ancient, and dated by *Relate* to be <100k YBP (see Materials and Methods). We confirm that signal strength is highly correlated (96%) to our IMI estimate for the high-coverage samples built into our *Relate* genealogy, where both estimates are available (supplementary fig. 12, Supplementary Material online). We do not observe the signal in Neanderthals (Prüfer et al. 2014, 2017) or Denisovans (Meyer et al. 2012), consistent with (Mathieson and Reich 2017). The signal appears already widespread in the Late Upper Paleolithic, as it is carried by Bichon (Western HG; 13.7k YBP), by Sidelkino (Eastern HG; 11k YBP), by SATP (Caucasus HG; 13k YBP), and Bon002 (Early Neolithic Anatolian; 10k YBP) (fig. 6*c*, supplementary fig. 13, Supplementary Material online).

We note that the Caucasus HG SATP has a strong signal, however, confidence intervals are large due to its lower coverage and this estimate may therefore be somewhat unreliable, although it seems clear that this individual carried the signal, which is also present in a later higher coverage Caucasus HG (KK1; 8k YBP). The Mal’ta individual (MA1) (Raghavan et al. 2014) has a similarly large confidence interval due to its lower coverage but may not have been a carrier of this signal. A 9,000 year-old Iranian farmer, WC1 (Broushaki et al. 2016), who can be modeled as a mixture of a “basal Eurasian” and Mal’ta-like ancestry, and who is not closely related to Anatolian farmers, likely only carried the signal weakly, if at all. Interestingly, Chan, a 9,000-year-old Iberian HG (Olalde et al. 2019) who has little ancestry related to Western HGs such as Bichon, and instead increased affinity to HGs predating these in Europe, has the weakest signal among all Mesolithic Europeans.

Already 10,000 years ago, the signal appears weaker in Western HGs compared with the Anatolian genome, who is among the strongest carriers of this signal (similar strength to later Neolithic individuals and present-day Sardinians) (fig. 6*e*), suggesting that the driver of this mutation rate change, which may have been of genetic or environmental nature, was already extinct by the Holocene. Eastern HGs have a slightly elevated signal compared with Western HGs. Overall, this provides direct support for previous analyses based on modern-day genomes that found a reduction of the TCC/TTC pulse to normal levels in the last 10 − 15k years (Speidel et al. 2019; DeWitt et al. 2021) and would imply that excess TCC/TTC mutations were subsequently passed on only through shared ancestry. Strikingly, the strength of the TCC/TTC signal shows a remarkable correlation with recent coalescence rates to the 10k-year-old Anatolian individual (96% using IMI for SGDP non-Africans and 13 high-coverage ancients, 71% using TCC/TTC proportion for ancients) (fig. 6*b* and *d*), and does not correlate as well with coalescence rates to any other HG group for whom we have data (88% or 58% with Caucasus HGs (SATP), 83% or 53% with Scandinavian HGs (sf12), 76% or 37% with Eastern HGs (Sidelkino), 73% or 53% with Western HGs (Bichon), where first number uses IMI, second number uses TCC/TTC proportion) (supplementary fig. 14, Supplementary Material online). We therefore hypothesize that the signal spread through ancestors of this Anatolian individual across Europe before the arrival of farming, and subsequently arrived in Europe for a second time with Neolithic farmers.

The genetic relationship among West Eurasian HG groups in the Late Paleolithic is not fully understood and, to the best of our knowledge, current models do not include a clear source group contributing widely across these HG groups, while able to explain the strong correlation to ancestry from Anatolia. One potential source are ancestors of the Dzudzuana Cave individuals, a group inhabiting the Caucasus ∼26k years ago (Lazaridis et al. 2018), from whom Anatolians are thought to derive the majority of their ancestry. This ancestry is present to a lesser extent in Caucasus HG and is even further diluted in Iranian Early Farmers. Dzudzuana-related populations may also have contributed ancestry to Eastern and Scandinavian HGs before the spread of farming. The Dzudzuana individuals have a pre-LGM common ancestor with Western HGs, including Bichon, however, placing the signal on this common ancestor lineage does not immediately explain the signal strength difference and correlation to shared ancestry with Anatolia. Two potential explanations include: The mutation rate elevation occurred in a Dzudzuana-like joint ancestor of Anatolian farmers and Western HGs, with subsequent dilution of the signal in Western HGs from an ancestry not closely related to Anatolia. Another possibility is that the mutation rate elevation occurred in a group more specific to Anatolia and that the signal spread during the Bølling-Allerød interstadial, a brief warming following the LGM, during which Western HGs spread across Europe replacing earlier HG groups and which may have introduced gene-flow from the Near East into Europe (Fu et al. 2016).

We note that although the cause of this mutation rate elevation remains uncertain, our results would fit well with a genetic cause within a specific ancient population (e.g., a mutation in some repair protein, transiently present). If, alternatively, the cause is environmental, it appears highly localized in both time and place, and this seems potentially harder to explain.

## Discussion

The last decade has seen an explosion in the number of sequenced ancient genomes, uncovering remarkable stories of population replacements and admixture that are associated with dramatic shifts in lifestyle arounds the world (Skoglund and Mathieson 2018). Although ancient genomes are still typically available in smaller numbers and lower quality compared with genomes of present-day people, they are uniquely valuable in providing direct insight into the genetic makeup of our ancestors. We have extended the *Relate* method for inference of genome-wide genealogies to work with ancient genomes and introduced a new method, *Colate*, for inference of coalescence rates for low-coverage unphased genomes. Together, these tools enable us to harness the power of genealogy-based analyses on a wider range of samples, including those of lower quality, which were previously inaccessible.

We demonstrated, using 278 moderns of the SGDP data set, 14 high-coverage, and 416 lower-coverage ancients, that *Relate* and *Colate* can uncover dynamic population histories and evolution in the processes that drive genetic variation. The extent to which directional gene-flow occurred from groups related to ancient Anatolia into European HGs predating the spread of farming in Europe has remained controversial. We have provided two further lines of evidence that such gene-flow existed, first using coalescence rates of lineages recently coalesced between Anatolia and HGs. The TCC/TTC mutation rate elevation in all these ancient groups, and its strong correlation to inferred recent shared ancestry with Anatolia, offers complementary support that the shared ancestry detected by *Colate* indeed reflects recent gene exchange, given the age distribution of samples showing this mutational phenomenon.

Future avenues of research may include using genealogies for parametric inference of population histories and admixture, inspired by approaches based on site-frequency spectra (Excoffier et al. 2013; Terhorst et al. 2017) and F-statistics (Patterson et al. 2012; Peter 2016; Ralph et al. 2020). Coalescence rates can be interpreted as a function of gene flow (or the lack thereof); for instance, Wang et al. (2020) have recently developed a method that infers migration rates through time given pairwise coalescence rate estimates. Genealogies of modern individuals have proven to be powerful in quantifying positive selection (Speidel et al. 2019; Stern et al. 2019, 2021) and genealogies including ancient genomes should further boost power.

Although *Colate* has made it possible to leverage genealogies for the study of low-coverage genomes possible, we ideally would like to incorporate such genomes directly into genealogical trees. This is currently not possible, however recent work building on the tsinfer methodology (Kelleher et al. 2019) provides an alternative approach that constrains the age of ancestral haplotypes using low-coverage ancient genomes to infer genome-wide genealogies for higher-quality phased sequences (incl. ancients and moderns) (Wohns et al. 2021). A possibility for making lower coverage ancient genomes, or indeed hybrid capture array data, accessible to these methods is imputation (Gamba et al. 2014; Hui et al. 2020; Rubinacci et al. 2021). A potential concern is that imputation may introduce biases, particularly in ancient genomes with ancestries that are not well reflected in modern groups. These biases are often difficult to assess. Because *Colate* does not require imputation, we expect that it will be a useful tool to investigate such biases in future.

## Materials and Methods

### Colate

Coalescence rates are inferred by attempting to maximize the following likelihood using an EM algorithm. For any derived mutation carried by a reference chromosome j, we ask whether this mutation is shared by the target chromosome i, which we denote by an indicator variable $S_{\ell ij}$ (ℓ indexing SNPs). We multiply across SNPs, such that no phase information is required to compute the likelihood. To obtain coalescence rates between groups of individuals, we also multiply the likelihood across homologous chromosomes in both the target and reference groups. To calculate within-individual coalescence rates using genotypes, the method assigns one allele to each category, at random at every SNP. When input is specified in BAM format (as reference-aligned reads), we multiply across reads. The maximum likelihood estimate is then given by $\hat{\theta }=\arg\underset{\theta }{\max}\prod_{\ell }\prod_{i,j}P\left(S_{\ell ij}\;\right|\;a_{\ell },\theta )$, where θ denotes piecewise-constant coalescence rates and $a_{\ell }$ is the age of the ℓth mutation, which we have to integrate out in practice.

To integrate out mutation age, we assume neutrality of every mutation, implying that its age is uniformly distributed on the branch onto which it maps. The EM algorithm requires us to integrate out mutation age conditional on sharing or not sharing between target and reference chromosomes. This theoretically implies a deviation from the uniform distribution. This deviation is strongest for mutations that are singletons in the genealogy used to date these mutations and are shared between sequences in the target and reference chromosome sets. In this case, knowledge of sharing implies that the mutation is older than the coalescence time of the target, reference, and corresponding individual in the genealogy, biasing mutation age upwards compared with a uniform distribution (supplementary fig. 15, Supplementary Material online). We use an empirical approach to sample mutation ages for these shared singletons and use the uniform distribution for all other mutations in practise, which we demonstrate is a reasonable approximation (supplementary information, Supplementary Material online). Moreover, we note that the *Colate* approach requires the inclusion of sites fixed and derived in all samples used for inferring the genealogy, as the additional reference and target samples can, in theory, coalesce into the root branch. To obtain an approximate upper bound on the age of such mutations, we fix the time to the most recent common ancestor (TMRCA) to an outgroup (10 M YBP for human-chimpanzee in this study).

We bin mutation ages into a discrete time grid to reduce computation time of the EM algorithm. As a result, the algorithm only requires the number of shared and not-shared mutations in each time grid as input; compilation of this input data is linear in sample size and number of mutations. Once in this form, the input data to the EM algorithm, and hence the computation time of the EM algorithm, is independent of sample size or the number of mutations.

### Simulations

To evaluate *Relate* and *Colate*, we used stdpopsim to simulate genomes with different demographic histories (Adrion et al. 2020) and hotspot recombination rates. For *Colate*, we additionally require an outgroup to determine mutations that are fixed in all samples. Instead of simulating an outgroup explicitly, we fixed the TMRCA $t_{\mathrm{out}}$ to the outgroup ($t_{\mathrm{out}}=10\;M\;\mathrm{years}$ in our simulations), and sampled the number of fixed mutations in any given region as a Poisson distributed random variable with mean $\mu l(t_{\mathrm{out}}-t_{\mathrm{sample}})$, where μ is the per base per generation mutation rate, $t_{\mathrm{sample}}$ is the TMRCA of the sample in this region and l is the number of base-pairs in this region. If $t_{\mathrm{sample}}$ was greater than $t_{\mathrm{out}}$, we sampled no fixed mutations. We then chose the base-pair positions of these fixed mutations uniformly at random with replacement within the corresponding region. For simplicity, we assumed a two-state mutation model, such that any occasional repeat mutation at one genomic site return to the original state.

Supplementary figure 2, Supplementary Material online shows the performance on a zigzag history (Schiffels and Durbin 2014), demonstrating near perfect recovery of coalescence rates when using true mutation ages in *Colate*, and high accuracy when mutation ages are sampled given a genealogy; the discrepancy highlights that our sampling distribution of mutation age given a genealogy (see Materials and Methods; supplementary information, Supplementary Material online) is reasonable but not exact.

We also simulated data under a multipopulation model of ancient Eurasia, previously fitted using real human genomes (Kamm et al. 2020), using the stdpopsim package. We simulated 200 haploid sequences in each of three modern human groups (Mbuti, Sardinian, Han), as well as four ancient Eurasians (LBK, Loschbour, Ust’-Ishim, MA1) and a Neanderthal (two haploid sequences in each group) (fig. 2*a*;supplementary fig. 3, Supplementary Material online). From this simulation, we obtained true genealogical trees and inferred *Relate* trees for all samples. In addition, we inferred a separate set of *Relate* trees using only the three modern human groups (Mbuti, Sardinian, Han), and used these to date mutations for *Colate*.

*Colate* recovered within and across group coalescence rates accurately compared with the corresponding direct MLEs calculated on true or Relate-inferred trees (fig. 2*a*).

Relate-inferred coalescence rates show that ancients coalesce with other individuals at the expected rates and in the correct order on average, as can be seen with MA1, for instance, who is inferred to have a shared history with LBK, Loschbour, and Sardinians, but form a population that splits off from Han around 50k years ago. In particular, these coalescence rates clearly captured the admixture from Neanderthals into an ancestral Eurasian lineage, as well as more recent genetic structure, such as separation of the Loschbour HG and early farmer lineages, represented by LBK. We observed a closer affinity of the Loschbour HG to modern-day Sardinians, compared with LBK, consistent with modern Sardinians being an admixture of HG and farmer ancestry in this simulation.

One case for which *Colate* performed less well compared with direct MLEs obtained from *Relate* trees is in inferring the cross-coalescence rates between Neanderthals and Mbuti, calculated by assigning the Neanderthal as reference and Mbuti as target. This is because the genealogy used to date mutations can provide dates only for variants segregating in the three modern groups. Therefore, the large majority of those Neanderthal sites that mutated more recently than the Neanderthal-Mbuti split cannot be used for inference. In this case, it would instead be preferable to assign Mbuti as reference.

### Evaluating *Colate* on Downsampled High-Coverage Genomes

We evaluated the performance of *Colate* on low-coverage sequencing data, by comparing estimates obtained from downsampled BAM files (fig. 2*b*, supplementary fig. 3, Supplementary Material online). To date mutations, we constructed a genealogy containing 25 diploid samples from each of the three 1000 Genomes populations—YRI (Yobura in Ibadan, Nigeria), CEU (Northern and Central European ancestry individuals from Utah, USA), and CHB (Han Chinese from Beijing, China) (1000 Genomes Project Consortium 2015), downloaded from http://ftp.1000genomes.ebi.ac.uk/vol1/ftp/release/20130502/. We then chose four 1,000 Genomes samples that were not incorporated into this genealogy as target individuals (HG00096, HG00268, NA18525, NA19017) and included the remaining samples in groups YRI, CEU, and CHB in the reference chromosomes set. The BAM files of these four genomes were obtained from ftp://ftp.1000genomes.ebi.ac.uk/vol1/ftp/technical/working/20140203_broad_high_cov_pcr_free_validation/matching_LC_samples_bwamem/ and subsequently downsampled to a variety of reduced sequencing coverages using SAMtools v1.9 (Li et al. 2009).

Across a wide range of mean coverages, *Colate*-inferred coalescence rates remained unchanged. To obtain 95% confidence intervals, we used a block bootstrap, dividing the genome into 20 Mb blocks, and resampling 100 times. Confidence intervals become wider for lower coverage sequencing data; encouragingly, we could infer meaningful coalescence rates between a target sequence of 0.01x mean coverage and the reference BCFs.

We additionally evaluated *Colate* when both target and reference samples are of low coverage by calculating the coalescence rates between LBK, a 7,200 year old early European farmer, and Loschbour, a nearly 8,000 year old Mesolithic Western HG (both >14x coverage) (Lazaridis et al. 2014) using a genealogy for SGDP to date mutations. We downsampled both individuals to a minimum of 0.1x mean coverage (supplementary fig. 4, Supplementary Material online). Although inference of coalescence rates became challenging when both genomes are at 0.1x, estimates still appeared reasonably accurate and unbiased.

### Data

#### SGDP Data

We downloaded phased haplotypes for 278 individuals from https://sharehost.hms.harvard.edu/genetics/reich_lab/sgdp/phased_data/PS2_multisample_public/, and rephased these jointly with high coverage ancients (Ancient Genomes Data) using SHAPEIT4 (Delaneau et al. 2019). We first used the 1000 Genomes Project (1000GP) reference panel (http://ftp.1000genomes.ebi.ac.uk/vol1/ftp/release/20130502/) to phase all sites overlapping with 1000GP and then internally phased all remaining sites, whereas keeping the already phased sites fixed. 

#### Ancient Genomes Data

We downloaded 430 ancient genomes for use in this study (supplementary table 1, Supplementary Material online). All samples had a genome-wide mean coverage of 0.5x or more. We selected 14 high coverage ancient genomes (mean genomic coverage >7.8x) for the *Relate* analysis.

For these 14 high coverage genomes (supplementary table 1, Supplementary Material online) genotypes were called using samtools mpileup (input options: -C 50, -Q 20 and -q 20) and bcftools call –consensus-caller with indels ignored (Li 2011). A modified version of the bamCaller.py script from https://github.com/stschiff/msmc-tools was used to output variant sites. We generated a quality mask for each ancient genome, declaring only sites with at least 5x coverage and below twice the mean genomic coverage as passing.

We merged these 14 ancient genomes with the 278 SGDP samples to infer joint genealogies using *Relate*. We constructed a conservative joint mask, declaring only sites passing in all of the 14 ancients, as well as a universal mask file provided with the SGDP data set, as passing. The SGDP universal mask was obtained from https://reichdata.hms.harvard.edu/pub/datasets/sgdp/filters/all_samples/.

### Joint Genealogies of Ancients and Moderns

We inferred joint genealogies of ancients and moderns using our updated *Relate* algorithm (supplementary information, Supplementary Material online). We used all mutations, excluding those in CpG contexts, to infer tree topologies and then restricted to transversion only for inference of branch lengths. Assuming an overall average mutation rate of 1.25 × 10^−8^ per base per generation and a transition to transversion ratio of 2 in humans (Ségurel et al. 2014), we therefore reduced the mutation rate for branch length inference to 4 × 10^−9^ per base per generation. We used a recombination map obtained from https://mathgen.stats.ox.ac.uk/impute/1000GP_Phase3.html and realigned alleles relative to an ancestral genome obtained from ftp://ftp.1000genomes.ebi.ac.uk/vol1/ftp/phase1/analysis_results/supporting/ancestral_alignments/. We otherwise used default parameters in *Relate*.

To infer branch lengths, we used a precomputed average coalescence rate estimate obtained by applying *Relate* to the 278 SGDP modern samples. To compute these coalescence rates, we jointly sampled branch lengths and effective population sizes using our updated iterative algorithm, which we show can be interpreted as an approximate EM algorithm for finding maximum likelihood coalescence rates. This approximate EM algorithm samples genealogies using *Relate* instead of integrating over all possible genealogies (see supplementary section B, Supplementary Material online). To obtain a coalescence rate estimate that matches the mutation rate used for inferring the genealogy of ancients and moderns, we inferred branch lengths using transversions only and set the mutation rate to 4 × 10^−9^ per base per generation.

### *Colate*-Inferred Coalescence Rates for SGDP and 430 Ancient Samples

We inferred coalescence rates for pairs of ancient individuals using *Colate*, restricting to transversions only. For each pair of samples, when given as a BCF file, we applied the respective mask files. When a sample was given in BAM file format, we accepted a read whenever its mapping quality exceeded 30, read length exceeded 34 bp, and there were fewer than three mismatching sites compared with the reference genome. We further excluded two base-pairs at each end of a read and restricted our analysis to sites where at most two different alleles were observed.

To date mutations, we used a *Relate*-inferred genealogy of the SGDP data set. As the degree of inbreeding varied across SGDP individuals (main text) and to avoid biases in mutation ages resulting from extensive inbreeding in some individuals, we selected one haploid sequence from each individual in constructing this genealogy. We jointly fitted branch lengths and coalescence rates using a mutation rate of 1.25 × 10^−8^ per base per generation.

### Inference of Directional Migration

To investigate evidence for directional migration, we focus on lineages that are recently coalesced (<50k YBP) between an individual in groups A and an individual in group B. If these groups split >50k YBP, then any such lineage should exclusively come from migrants of one group to the other, or at least should in practice be highly enriched for such migrant lineages. Therefore, if migration occurred purely from group A into group B, these recently coalesced lineages can be classified as belonging to group A back in time and should behave like any other lineage in group A. The approach is expected to also be robust to earlier split times (<50k YBP), because lineages behave identically once groups have merged, reflected in identical coalescence rates for epochs predating the split.

We test this by calculating coalescence rates of such recently coalesced lineages to each non-African SGDP individual, integrated between 0 and 50k YBP and stored in variable y. We expect that these coalescence rate profiles differ between lineages assigned to groups A and B and we can distinguish whether a lineage belongs to either group. We fit this (N×1 vector, N being the number of non-African SGDP individuals) vector y as a mixture of coalescence rates (also integrated between 0 and 50k YBP) of k surrogate source individuals to non-African SGDP individuals, denoted by $x_{k}$ (N×1 vectors). We use non-negative least squares, such that the coefficients β (k×1 vector) are given by

$$\hat{\beta }=\mathrm{argmi}n_{\beta \geq 0}{\left\|y-X\beta \right\|}_{2},$$

where ${\left\|\right\|}_{2}$ denotes the Euclidean norm and X is a N×k matrix with columns given by $x_{k}$. We use the *R* function nnls to find nonlinear least squares estimates and bootstrap entries of our vectors y and $x_{k}$ to obtain confidence intervals.

### Calculation of Mutation Rate

We calculated mutation rates for 76 mutation triplets (of 4 × 4 × 3/2 = 96 possible) in each individual, after excluding any singletons and terminal branches in our genealogy. We only considered mutation triplets that are not in a CpG context, which excludes 20 possible triplets. To remove trends shared across mutation triplets, we divided the TCC/TTC mutation rate by the average over all triplets (excl. CpG contexts) in each epoch, to obtain the mutation rate relative to the average mutation rate.

To calculate the area under the curve for the TCC/TTC mutation rate signature, we first scaled the mutation rate for this triplet in each individual by the average across triplets over the time interval [1e5,1e6] YBP (predating the emergence of this signature). We then calculated the IMI, which is the area under the curve between 14k and 1 My BP, where time is measured in log10 units to upweight the recent past. For samples that are older than 14k years (Ust’-Ishim, Sunghir3, and Yana1), we extrapolated the earliest value to 14k YBP. We then subtracted the equivalent value of a constant mutation rate from this IMI, such that any sample without the elevation in TCC/TTC mutation rates is expected to have an IMI of 0.

### Quantifying the TCC/TTC Signal in Lower Coverage Individuals

We quantified the TCC/TTC signal in lower coverage individuals (>2x mean coverage) by restricting to sites segregating in our SGDP genealogy that we also used to date mutation in *Colate*. We additionally restricted to sites where the age of the upper coalescence event of the branch onto which the mutation maps is <100k YBP. For each sample, at any such site, we then further restricted to sites where there were at least four reads mapping and added a count toward a mutation category in that individual if at least four reads supported the derived allele. In this way, we counted the number of sites with strong evidence of being in a heterozygous or homozygous state for the derived allele. We finally calculated the proportion of such sites, relative to any C/T transitions, excluding those in CpG context. We calculated confidence intervals using a block bootstrap with block size of 10 Mb.

Ascertainment of mutations in moderns may potentially downwards bias signal strength in some ancients, if these possess private TCC/TTC variants less likely to be transmitted to modern individuals compared with other transitions. This could happen for instance if close ancestors of an individual carried the driver of this mutation rate pulse generating private variants. However, regardless, we still expect this approach to find the group from which the signal spread into modern-day humans. In addition, the overall good agreement with the IMI estimates obtained from *Relate* genealogies of high-coverage samples (supplementary fig. 12, Supplementary Material online), where no such ascertainment is done, we believe that any such biases have only a minor effect.

### Calculation of Pairwise F2 Statistics

We calculated F2 statistics between ancients for comparisons to matrices of pairwise coalescence rates (used in supplementary fig. 7, Supplementary Material online). To calculate F2 statistics, we first made pseudohaploid calls for each individual using “pileupcaller” (https://github.com/stschiff/sequenceTools), where we restricted to 1240k ascertained genomic sites known to be varying among present-day humans (Mathieson et al. 2015). We then merged individuals using “mergeit” (https://github.com/DReichLab/EIG). To calculate F2 statistics, we used the R package admixtools2 (https://github.com/uqrmaie1/admixtools).

## Supplementary Material

Supplementary data are available at *Molecular Biology and Evolution* online.

## Supplementary Material

msab174_Supplementary_Data
