# Supplementary material for: Inferring Population Histories for Ancient Genomes Using Genome-Wide Genealogies
Source: Mol Biol Evol. 2021 Jun 15;38(9):3497–511. doi: 10.1093/molbev/msab174 (PMC8383901; doi:10.1093/molbev/msab174)
Supplement: msab174_Supplementary_Data [file msab174_supplementary_data.zip › Colate_MBE_SI.pdf]

Supplementary Information:
Inferring population histories for ancient genomes using genome-wide genealogies

Leo Speidel, Lara Cassidy, Robert W. Davies,
Garrett Hellenthal, Pontus Skoglund, Simon R. Myers

A. Colate **1**

A.1 Notation 1

A.2 Overview of the Colate method 2

A.3 Expectation-Maximisation algorithm with known mutation ages and genotypes 2

A.4 Expectation-Maximisation algorithm with unknown mutation ages 4

A.5 Sampling mutation ages given genealogical constraints 5

B. Relate: Approximate EM algorithm for inferring coalescence rates from a genealogy **7**

C. Adapting Relate to build genealogies including ancient genomes **9**

Supplementary Figures ***12***

A. Colate

A.1 Notation

We will use the following notation in this section:

- e indexes epochs
- τ_e denotes the lower boundary of epoch e
- $\theta(t)$ denotes coalescence rates through time and takes the form $\theta(t) = \sum_{e=1}^{E} \theta_e \, 1_{\tau_{e-1} \leq t < \tau_e}$, where 1 is the indicator function. We write $\boldsymbol{\theta} = (\theta_e)_{e=1,\dots,E}$.
- S_ℓ is the indicator of sharing/non-sharing of a mutation at site ℓ
- t_ℓ is the coalescence time at SNP $\boldsymbol{\ell}$**,** which is unknown

- a_ℓ is the age of a mutation at site ℓ and l_ℓ and u_ℓ are the lower and upper times of the branch onto which this mutation maps.

A.2 Overview of the Colate method

Throughout, we assume to have one reference and one target sequence. In cases where we have multiple reference or target sequences (e.g., in non-haploid organisms, or groups of individuals), we use a composite likelihood approach and multiply the likelihood across individuals. Colate can be applied to reference-aligned read data directly by constructing a composite likelihood that multiplies over reads. We also use a composite likelihood approach across genomic sites and therefore require no phase information for non-haploid organisms.

Epoch boundaries τ_e are prespecified parameters and we assume that the coalescence rate is given by a piecewise-constant function $\theta(t) = \sum_{e=1}^{E} \theta_e \, 1_{\tau_{e-1} \leq t < \tau_e}$. We aim to find a maximum likelihood estimate of the coalescence rates $\boldsymbol{\theta} = (\theta_e)_{e=1,\ldots,E}$.

For any mutation carried by the reference sequence, we observe whether the mutation is also carried by the target sequence. This is our observed data and is stored in the indicator variable S_ℓ equaling 1 if mutation ℓ is shared and 0 if it is not shared. In the following Expectation-maximisation (EM) algorithm, the coalescence time t_ℓ at SNP ℓ between the target and the reference sequence is the unobserved latent variable which we will integrate out. In the first part, we will assume that mutation age a_ℓ is known and we will extend our method to the case when mutation age is unknown in the second part. The EM algorithm maximises $\prod_\ell P(S_\ell \mid a_\ell, \boldsymbol{\theta})$ (ℓ indexing SNPs) with respect to coalescence rates $\boldsymbol{\theta}$, outputting an approximate maximum likelihood estimate (MLE) $\widehat{\boldsymbol{\theta}}$. We obtain uncertainty estimates around this MLE using a block bootstrap on genomic regions.

A.3 Expectation-Maximisation algorithm with known mutation ages and genotypes

We assume that mutation ages a_ℓ are known. Then, the loglikelihood of $\boldsymbol{\theta}$ given the data S_ℓ and latent variable t_ℓ is

$$\log P(S_\ell, t_\ell \mid a_\ell, \boldsymbol{\theta}) = \log P(S_\ell \mid t_\ell, a_\ell, \boldsymbol{\theta}) + \log f(t_\ell \mid a_\ell, \boldsymbol{\theta}), \tag{1}$$

where $P(S_\ell \mid t_\ell, a_\ell, \boldsymbol{\theta})$ is a step function given by

$$P(S_\ell = 1 \mid t_\ell, a_\ell, \boldsymbol{\theta}) = \begin{cases} 1 & \text{, if } t_\ell \leq a_\ell \\ 0 & \text{, otherwise} \end{cases} \tag{2}$$

$$P(S_\ell = 0 |\, t_\ell, a_\ell, \boldsymbol{\theta}) = \begin{cases} 1 & \text{, if } t_\ell > a_\ell \\ 0 & \text{, otherwise.} \end{cases}$$

This step function reflects our infinite-sites assumption: a mutation can only be shared if it is older than the time to the most recent common ancestor (TMRCA) between the target and reference sequence and it can only be not shared if it is younger than the TMRCA. In particular, this step function does not depend on $\boldsymbol{\theta}$. The density of coalescence time t_ℓ is a non-homogeneous exponential given by the standard coalescent, which does not depend on mutation age a_ℓ assuming neutrality; it is therefore given by

$$\log f(t_\ell \mid a_\ell, \boldsymbol{\theta}\,) = \log \theta(t_\ell) - \int_0^{t_\ell} \theta(s)\, ds\,. \tag{3}$$

By using that $\theta(t) = \sum_{e=1}^{E} \theta_e \, 1_{\tau_{e-1} \le t < \tau_e}$, we can rewrite Eq. (3) as

$$\log f(t_\ell \,|a_\ell, \boldsymbol{\theta}\,) = \sum_e \log \theta_e \; 1_{\tau_{e-1} \le t_\ell < \tau_e} - \sum_e \theta_e \big[(t_\ell - \tau_{e-1}) 1_{\tau_{e-1} \le t_\ell < \tau_e} + (\tau_e - \tau_{e-1}) 1_{t_\ell \ge \tau_e} \big], \tag{4}$$

where 1_X denotes the indicator function equaling one if and only if X is true and 0 otherwise.

The EM-algorithm requires us to integrate out the latent variable t_ℓ conditional on the data and the coalescence rates of the previous iteration, denoted by $\boldsymbol{\theta}^{(\boldsymbol{k})}$. Substituting Eq. (4) in Eq. (1) and taking the expectation, we obtain

$$\begin{aligned} E_{t_\ell}&\big[\log P(\, S_\ell, t_\ell \mid a_\ell, \boldsymbol{\theta}\,) \mid S_\ell, a_\ell, \boldsymbol{\theta}^{(k)}\big] \\ &= \; const + \sum_e \log \theta_e \;\; P(\tau_{e-1} \le t_\ell < \tau_e \mid S_\ell, a_\ell, \boldsymbol{\theta}^{(\boldsymbol{k})}) \\ &- \sum_e \theta_e \left[\int_{\tau_{e-1}}^{\tau_e} (s - \; \tau_{e-1})\, f(s |\, I_\ell, a_\ell, \boldsymbol{\theta}^{(\boldsymbol{k})})\, ds + \; (\tau_e - \; \tau_{e-1})\, P(t_\ell \ge \; \tau_e \mid \; S_\ell, a_\ell, \boldsymbol{\theta}^{(\boldsymbol{k})}) \right]. \end{aligned} \tag{5}$$

Equation (5) is the expected log-likelhood for one SNP. We use a composite likelihood across SNPs, such that the expected log-likelihood genome-wide is a sum of Eq. (5) across all SNPs. To complete the EM update, we maximise the expected loglikelihood with respect to $\boldsymbol{\theta}$ to obtain our updated estimate $\boldsymbol{\theta}^{(\boldsymbol{k+1})}$**.** By finding the root of the first derivative with respect to θ_e, we obtain

$$\theta_e^{(k+1)} = \frac{\sum_\ell P(\tau_{e-1} \leq t_\ell < \tau_e \mid S_\ell, a_\ell, \boldsymbol{\theta}^{(k)})}{\sum_\ell \int_{\tau_{e-1}}^{\tau_e} (t_\ell - \tau_{e-1})\, f(t_\ell \mid S_\ell, a_\ell, \boldsymbol{\theta}^{(k)})\, dt_\ell + (\tau_e - \tau_{e-1})\, P(t_\ell \geq \tau_e \mid S_\ell, a_\ell, \boldsymbol{\theta}^{(k)})}. \tag{6}$$

The numerator of Eq. (6) is the probability that the coalescence event occurred in epoch e. The denominator of Eq. (6) is the opportunity (or expected branch length) of the coalescence event happening in epoch e. Evaluation of Eq. (6) requires calculating integrals of $f(t_\ell \mid S_\ell, a_\ell, \boldsymbol{\theta}^{(k)}) \propto P(S_\ell \mid t_\ell, a_\ell, \boldsymbol{\theta}^{(k)})\, f(t_\ell \mid a_\ell, \boldsymbol{\theta}^{(k)})$, which is given by Eqs. (1)-(3) and is effectively an integral of the exponential prior density of coalescence times over an adjusted domain that excludes coalescence events incompatible with the data (i.e., sharing/non-sharing of the mutation).

In practice, we use a discrete time grid to calculate Eq. (6). By doing so, we can bin SNPs by age bins, such that we only have to calculate a constant number of integrals (not growing with the number of SNPs) to evaluate Eq. (6).

When multiple target and/or reference sequences are used, we precompute the how often the mutation is shared and non-shared by age bin. Given these precomputed values, evaluation of Eq. (6) is not dependent on the number of SNPs or the number of target and reference sequences. Counting how often a mutation is shared and non-shared only requires computing the derived allele frequencies in the target and reference sample and is given by $f_\ell^t f_\ell^r$ and $(N^t - f_\ell^t) f_\ell^r$, respectively, where f_ℓ^* denotes the derived allele frequency and N^* the number of sequences.

Overall, the computational complexity of this EM algorithm is constant with respect to number of SNPs and number of sequences, beyond calculating the number of shared/non-shared mutations by age bin, which itself takes linear time (in number of SNPs and number of sequences) and requires little computation beyond parsing the data and computing derived allele frequencies.

A.4 Expectation-Maximisation algorithm with unknown mutation ages

In practice, mutation ages are unknown and we infer mutation ages using a genealogy. This genealogy is inferred for individuals that may be distinct from the reference sequences in the EM algorithm, e.g., in practice, we might use a large sample to infer a genealogy to date mutations, and subsequently infer coalescence rates between targets and a subset of the sequences used to infer the genealogy, or two target sequences. A genealogy will limit the mutation age a_ℓ to a range between the lower and upper boundaries of the branch onto which the mutation maps, which we denote by l_ℓ and u_ℓ. We modify our EM algorithm and treat mutation age as an additional latent variable, in addition to the coalescence time t_ℓ, such that Eq. (1) is updated to

$$\log P(S_\ell, t_\ell, a_\ell \mid l_\ell, u_\ell, \boldsymbol{\theta}) = \log P(S_\ell \mid t_\ell, a_\ell, l_\ell, u_\ell, \boldsymbol{\theta}) + \log f(t_\ell \mid a_\ell, l_\ell, u_\ell, \boldsymbol{\theta}) + \log f(a_\ell \mid l_\ell, u_\ell, \boldsymbol{\theta}). \tag{7}$$

Here, $P(S_\ell \mid t_\ell, a_\ell, l_\ell, u_\ell, \boldsymbol{\theta})$ is still the same step function and does not depend on l_ℓ, u_ℓ, and $\boldsymbol{\theta}$. The density of mutation ages $f(a_\ell \mid l_\ell, u_\ell, \boldsymbol{\theta})$ is given by the uniform distribution between l_ℓ and u_ℓ and does not depend on $\boldsymbol{\theta}$. We note that $f(t_\ell \mid a_\ell, l_\ell, u_\ell, \boldsymbol{\theta})$ is no longer given by a non-homogeneous exponential, as we are conditioning on l_ℓ and u_ℓ (by which we are conditioning on aspects of the local genealogy at this position).

Using Eq. (7), the expected log-likelihood is given by

$$\begin{aligned} &E_{a_\ell, t_\ell}\left[\log P(S_\ell, t_\ell, a_\ell \mid l_\ell, u_\ell, \boldsymbol{\theta}) \mid S_\ell, l_\ell, u_\ell, \boldsymbol{\theta}^{(k)}\right] \\ &\qquad = const + \int_{l_\ell}^{u_\ell} E_{t_\ell}\left[\log f(t_\ell \mid a_\ell, l_\ell, u_\ell, \boldsymbol{\theta}) \mid a_\ell, S_\ell, l_\ell, u_\ell, \boldsymbol{\theta}^{(\boldsymbol{k})}\right] f(a_\ell \mid S_\ell, l_\ell, u_\ell, \boldsymbol{\theta}^{(\boldsymbol{k})})\, da_\ell. \end{aligned} \tag{8}$$

Instead of evaluating the integral over a_ℓ, we will attempt to sample a_ℓ from the distribution $f(a_\ell \mid S_\ell, l_\ell, u_\ell, \boldsymbol{\theta}^{(\boldsymbol{k})})$. If we can sample a_ℓ in an unbiased way, we on average "know" the age of the mutation and can approximate Eq. (8) using

$$E_{a_\ell, t_\ell}\left[\log P(S_\ell, t_\ell, a_\ell \mid l_\ell, u_\ell, \boldsymbol{\theta}) \mid S_\ell, l_\ell, u_\ell, \boldsymbol{\theta}^{(k)}\right] \approx const + \frac{1}{M}\sum_{a'} E_{t_\ell}\left[\log f(t_\ell \mid a'_\ell, \boldsymbol{\theta}) \mid a'_\ell, S_\ell, \boldsymbol{\theta}^{(\boldsymbol{k})}\right], \tag{9}$$

where the sum goes over sampled mutation ages a' and M denotes the number of sampled ages. Because the expected log-likelihood across the entire genome is a sum over mutations, this brings us back to the case where mutation age is known.

A.5 Sampling mutation ages given genealogical constraints

It is key to sample from the conditional density of allele age $f(a_\ell \mid S_\ell, l_\ell, u_\ell, \boldsymbol{\theta}^{(\boldsymbol{k})})$ in an unbiased way. Here we illustrate an approximate approach that works well in practice. We use Bayes' theorem and obtain

$$\begin{aligned} f(a_\ell \mid S_\ell, l_\ell, u_\ell, \boldsymbol{\theta}^{(\boldsymbol{k})}) &\propto P(S_\ell \mid a_\ell, l_\ell, u_\ell, \boldsymbol{\theta}^{(\boldsymbol{k})})\, f(a_\ell \mid l_\ell, u_\ell, \boldsymbol{\theta}^{(\boldsymbol{k})}) \\ &= P(S_\ell \mid a_\ell, l_\ell, u_\ell, \boldsymbol{\theta}^{(\boldsymbol{k})})\, f(a_\ell \mid l_\ell, u_\ell) \\ &= \frac{P(S_\ell \mid a_\ell, l_\ell, u_\ell, \boldsymbol{\theta}^{(\boldsymbol{k})})}{u_\ell - l_\ell}, \end{aligned} \tag{10}$$

where we use that unconditionally, the age of a mutation is uniformly distributed between l_ℓ and u_ℓ. We are therefore interested in the functional form of $P(S_\ell \mid a_\ell, l_\ell, u_\ell, \boldsymbol{\theta}^{(\boldsymbol{k})})$. We argue that

$$P(S_\ell \mid a_\ell, l_\ell, u_\ell, \boldsymbol{\theta}^{(\boldsymbol{k})}) \approx P(S_\ell \mid l_\ell, u_\ell, \boldsymbol{\theta}^{(\boldsymbol{k})}), \tag{11}$$

where the right-hand side does not depend on mutation age a_ℓ, implying that Eq. (10) is the uniform distribution on $[l_\ell, u_\ell)$. Intuitively, this means that the probability of sharing (or non-sharing) does not depend on the mutation age, beyond conditioning on boundaries of the branch it falls on; this should be accurate if the probability of coalescing into this branch is negligible, and the more likely scenario is that coalescences happen either before l_ℓ or after u_ℓ. We show that empirically, this is the case in Supplementary Figure 15.

As Supplementary Figure 15 shows, approximating $f(a_\ell \mid S_\ell, l_\ell, u_\ell, \boldsymbol{\theta}^{(\boldsymbol{k})})$ by the uniform distribution is reasonable in most cases. An important exception are mutations that appear as singletons among samples used to infer the genealogy but which are carried by the target and reference sequences. We will refer to these mutations as "shared singletons". The age of a shared singleton is not well approximated by a uniform distribution, because the target and reference sequence coalescence into the branch onto which this singleton maps with certainty.

We therefore treat shared singletons separately by sampling from the following empirical distribution of singleton age. For shared singletons, we therefore approximate the distribution function of its age a by

$$\begin{aligned} F(t) = P(a \leq t \mid S_\ell = 1, \boldsymbol{\theta}^{(\boldsymbol{k})}) \propto P(S_\ell = 1 \mid a \leq t, \boldsymbol{\theta}^{(\boldsymbol{k})})\, P(a \leq t \,\big|\, \boldsymbol{\theta}^{(\boldsymbol{k})}) \\ \approx P(S_\ell = 1 \mid \text{upper boundary} \leq t) \frac{t}{const}. \end{aligned} \tag{12}$$

We calculate $P(S_\ell = 1 \mid \text{upper boundary} \leq t)$ empirically using the fraction of shared singletons with upper boundary not greater than t. The term $t/const$ assumes that a mutation happens sometime between time 0 and the time to the shared ancestor with an outgroup, such that unconditionally of sharing/non-sharing, the distribution of the age of a singleton is approximately uniform. Using Eq. (12), we can now sample the age of a singleton conditional on whether it is shared, using the inverse-transform trick, such that $a \sim F^{-1}(U)$, with U being a uniform random variable on [0,1].

B. Relate: Approximate EM algorithm for inferring coalescence rates from a genealogy

In (Speidel et al. 2019), we described an iterative algorithm for estimating branch lengths and coalescence rates; this algorithm iteratively inferred maximum likelihood coalescence rates given a tree, and then used these coalescence rates to reestimate branch lengths. This algorithm worked well in practise, but was heuristic.

Here, we describe how a minor modification of this algorithm can be interpreted as a Monte-Carlo EM (Caffo et al. 2005) that attempts to find the maximum likelihood coalescence rates for given data, essentially integrating out the possible genealogical histories by sampling these using Relate.

As before, we let $\boldsymbol{\theta} = (\theta_e)_{e=1,\ldots,E}$ be the coalescence rates in epochs $e = 1, \ldots, E$. Here, we describe a method that is slightly modified from the method in Speidel et al. (2019) for inferring coalescence rates using genealogies. We aim to find the maximum likelihood estimate

$$\widehat{\boldsymbol{\theta}} = \arg\max P(\boldsymbol{D} \mid \boldsymbol{\theta}) \; = \arg\max \int P(\boldsymbol{D}, \boldsymbol{T} \mid \boldsymbol{\theta}) \, d\boldsymbol{T}\,, \tag{13}$$

where $\boldsymbol{D}$ is the observed genetic variation data and $\boldsymbol{T} = (T_\ell)_{\boldsymbol{\ell}}$ is the collection of local genealogies, which we treat as unobserved latent variables in the following EM algorithm. For one marginal tree T_ℓ the log likelihood is given by

$$\log P(\boldsymbol{D}, T_\ell \mid \boldsymbol{\theta}\,) = \log P(\boldsymbol{D} \mid T_\ell) + \log f(T_\ell \mid \boldsymbol{\theta}), \tag{14}$$

where $P(\boldsymbol{D} \mid T_\ell)$ is typically given by a Poisson model (mutations happening at a constant rate μ), which does not depend on coalescence rates $\boldsymbol{\theta}$, and $f(T_\ell \mid \boldsymbol{\theta})$ is the coalescent prior of the marginal tree given coalescence rates. Denoting our estimate of the coalescence rate in step k of the EM algorithm by $\boldsymbol{\theta}^{(\boldsymbol{k})}$ and multiplying likelihoods across trees, the update of the EM algorithm is given by

$$\boldsymbol{\theta}^{(\boldsymbol{k+1})} = \arg\max \sum_{\boldsymbol{\ell}} E_{T_\ell}\big[\log f(T_\ell \mid \boldsymbol{\theta}) \mid \boldsymbol{D}, \boldsymbol{\theta}^{(k)}\big]\,. \tag{15}$$

Integrating formally over marginal trees given the data is difficult, so instead we use genealogies sampled by Relate, such that if we sample M trees per locus, we obtain

$$\boldsymbol{\theta}^{(\boldsymbol{k+1})} = \arg\max \frac{1}{M} \sum_{\boldsymbol{\ell}} \sum\nolimits_{\boldsymbol{T_\ell} \text{ sampled from } f(\boldsymbol{T_\ell} \mid \boldsymbol{D}, \boldsymbol{\theta}^{(\boldsymbol{k})})} \log f(T_\ell \mid \boldsymbol{\theta})\,. \tag{16}$$

In this approach, tree topology is fixed, and branch lengths are sampled from the posterior distribution given the data (mutations mapped to branches).

We note that in practise we only sample one set of branch lengths per marginal tree ($M = 1$). While we expect this to work well because we are integrating over many loci genome-wide, such that we capture enough variation in local genealogies in Eq. (16), we also note that our current implementation allows sampling of multiple branch lengths by appending the same chromosome repeatedly. In Speidel et al. (2019), where the algorithm for estimating coalescence rates was formulated in a more heuristic way, we instead used posterior mean branch lengths; by sampling branch lengths the algorithm is now an approximate EM algorithm.

Another difference to Speidel et al. (2019) is that we use the full coalescent prior in our approach here, whereas we used the coalescent prior for two haploid sequences in Speidel et al. (2019) and then averaged over all pairs of haploid sequences afterwards. Denoting by t_j the time of the coalescence event reducing the number of lineages from $j+1$ to j back in time, such that $t_{j+1} < t_j$, the coalescent prior is given by

$$f(T_\ell \mid \boldsymbol{\theta}) = \prod_{j=2}^{N} \binom{j}{2} \, \theta(t_j) \, e^{-\binom{j}{2} \int_{t_{j+1}}^{t_j} \theta(s)\, ds}, \tag{17}$$

where coalescence rates are piecewise constant, i.e., $\theta(t) = \sum_{e=1}^{E} \theta_e \, 1_{\tau_{e-1} \le t < \tau_e}$. Applying this to the logarithm of Eq. (17), we obtain

$$\begin{aligned} \log f(T_\ell \mid \boldsymbol{\theta}) = \sum_{j=2}^{N} \Bigg[& \log \binom{j}{2} + \sum_e \log \theta_e \; 1_{\tau_{e-1} \le t_j < \tau_e} - \binom{j}{2} \sum_e \theta_e \big(t_j - \max(\tau_{e-1}, t_{j+1})\big) 1_{\tau_{e-1} \le t_j < \tau_e} \\ & - \binom{j}{2} \sum_e \theta_e \big(\tau_e - \max(\tau_{e-1}, t_{j+1})\big) 1_{\, t_{j+1} < \tau_e,\, t_j \ge \tau_e} \Bigg]. \end{aligned} \tag{18}$$

Substituting Eq. (18) in Eq. (15) and assuming that we only sample one marginal tree per locus (which is the case in practice), we obtain

$$\begin{aligned} \boldsymbol{\theta}^{(\boldsymbol{k+1})} = \arg\max \sum_{\boldsymbol{\ell}} \sum_{j=2}^{N} \Bigg[& \sum_e \log \theta_e \; 1_{\tau_{e-1} \le t_{\ell,j} < \tau_e} - \binom{j}{2} \sum_e \theta_e \big(t_{\ell,j} - \max(\tau_{e-1}, t_{\ell,j+1})\big) 1_{\tau_{e-1} \le t_{\ell,j} < \tau_e} \\ & - \binom{j}{2} \sum_e \theta_e \big(\tau_e - \max(\tau_{e-1}, t_{\ell,j+1})\big) 1_{\, t_{\ell,j+1} < \tau_e,\, t_{\ell,j} \ge \tau_e} \Bigg], \end{aligned} \tag{19}$$

where $t_{\ell,j}$ denotes the coalescence time of the event reducing the number of lineages from $j+1$ to j in the ℓth tree. Calculating the root of the first derivative with respect to θ_e gives

$$\theta_e^{(k+1)} = \frac{\sum_{\ell} \sum_{j=2}^{N} 1_{\tau_{e-1} \leq t_{\ell,j} < \tau_e}}{\sum_{\ell} \sum_{j=2}^{N} \left[\binom{j}{2} \left(\left(t_{\ell,j} - \max(\tau_{e-1}, t_{\ell,j+1})\right) 1_{\tau_{e-1} \leq t_{\ell,j} < \tau_e} + \left(\tau_e - \max(\tau_{e-1}, t_{\ell,j+1})\right) 1_{t_{\ell,j+1} < \tau_e,\, t_{\ell,j} \geq \tau_e} \right) \right]}. \tag{20}$$

Similarly to Eq. (6), the numerator of Eq. (20) counts the number of coalescence events happening in epoch e and the denominator of Eq. (20) measures the total opportunity for a coalescence event in this epoch. We note that if $N = 2$, $t_{\ell,2}$ is the coalescence time between two sequences and $t_{\ell,3} = 0$, such that Eq. (20) reduces to the estimator derived in Speidel et al. (2019) for two haploid sequences.

C. Adapting Relate to build genealogies including ancient genomes

We modified the tree builder for constructing tree topologies and the branch length sampling scheme in our Relate method; the remainder of the method is unchanged and we refer the reader to Ref. (Speidel et al. 2019) for details of the method.

Tree builder for ancient genomes

The challenge with including ancient genomes is that these impose hard constraints on branch lengths and coalescence times; any coalescence event has a minimum age which is the maximum age of its descendants. We therefore modified the tree builder to discourage coalescences between contemporary and non-contemporary genomes when there is no strong evidence for this coalescence.

To do this, we calculate a preliminary date for coalescence events while inferring tree topology. Our tree builder constructs local genealogical trees bottom-up, so in each step of the tree builder, we have a certain number of lineages k left from which we select two to coalesce. To preliminarily date events, we use the expected time while k lineages remain in the coalescent model with a pre-specified effective population size N_e, which is given by $N_e / \binom{k}{2}$. In every step of our tree building algorithm, we identify potential lineages that we can coalesce, using the identical approach taken in our original approach (Speidel et al. 2019). Whenever multiple candidates arise, we exclude coalescences between non-contemporary samples and other lineages, if the age of that lineage is less than the sampling age of the non-contemporary sample. However, if the only possible coalescences are between

disallowed lineages, we allow such coalescences. This procedure therefore discourages coalescing contemporary and non-contemporary lineages unless there is evidence to do so.

Identification of feasible coalescence events is identical to before, where we find pairs of lineages that are mutually minimal in a non-symmetric distance matrix calculated using a modified chromosome painting hidden Markov model (Li and Stephens 2003; Speidel et al. 2019). Whenever we have more than one feasible pair of lineages (that are allowed to coalesce in our rule for non-contemporary samples above), we choose the pair with minimal distance in the symmetrised distance matrix.

Markov-chain Monte Carlo sampler for branch lengths

We modified the MCMC update rules to allow for non-contemporary samples. This MCMC algorithm samples from the following posterior distribution

$$\begin{aligned} &P(\text{branch lengths} \mid \text{tree topology}, \text{mutations}, \boldsymbol{\theta}) \\ &\qquad \propto P(\text{mutations} \mid \text{branch lengths}\ )\, f(\text{branch lengths} \mid \text{tree topology}, \boldsymbol{\theta}), \end{aligned} \tag{21}$$

where $P(\text{mutations} \mid \text{branch lengths}\ )$ is the likelihood function given by a Poisson model with a constant mutation rate and $f(\text{branch lengths} \mid \text{tree topology}, \boldsymbol{\theta})$ is the coalescent prior on branch lengths.

We have two ways of proposing new branch lengths, which are chosen at random with probability 0.4 and 0.6, respectively.

Swapping the times of two events (identical to existing Relate approach)

This step is unchanged. We choose two events at random and propose a switch of their coalescence times, if this does not violate tree topology. The times while k lineages remain are unchanged and the update step only requires recalculation of the likelihood function of the six branches that have been proposed to change in length (two daughter and one parent branch for each of the two events).

Update a single event between older daughter and parent (new)

For modern samples, we previously used an update step that proposed a new time for the time while k ancestors remain. Here, we replace this step with a new update step that proposes to only change the timing of one

coalescence event to any time between its older daughter event and parent event. We first choose one coalescence event at random. Defining the age of the older daughter coalescence event by t_{d} and the age of the parent coalescence event by t_{p}, the proposed age of the chosen event is drawn from a uniform distribution on $[t_{\mathrm{d}}, t_{\mathrm{p}})$.

The acceptance probability in a Metropolis-Hastings type MCMC sampler is given by the ratio of proposal probabilities of the old and new age of the chosen coalescence event, multiplied by the ratio of posterior probabilities of the old and new branch lengths. Conveniently, the proposal distribution is symmetric with respect to old and new ages, such that the ratio for the proposal probabilities is 1. It remains to evaluate the ratio of posterior probabilities of branch lengths, which are given by Eq. (21).

References

Adrion JR, Cole CB, Dukler N, Galloway JG, Gladstein AL, Gower G, Kyriazis CC, Ragsdale AP, Tsambos G, Baumdicker F, et al. 2020. A community-maintained standard library of population genetic models. *Elife* 9:e54967.

Caffo BS, Jank W, Jones GL. 2005. Ascent-based Monte Carlo expectation- maximization. *J. R. Stat. Soc. Ser. B (Statistical Methodol.*

Kamm J, Terhorst J, Durbin R, Song YS. 2020. Efficiently Inferring the Demographic History of Many Populations With Allele Count Data. *J. Am. Stat. Assoc.* 115:1472–1487.

Li N, Stephens M. 2003. Modeling Linkage Disequilibrium and Identifying Recombination Hotspots Using Single-Nucleotide Polymorphism Data. *Genetics* 165:2213–2233.

Speidel L, Forest M, Shi S, Myers SR. 2019. A method for genome-wide genealogy estimation for thousands of samples. *Nat. Genet.* 51:1321–1329.

Supplementary Figures

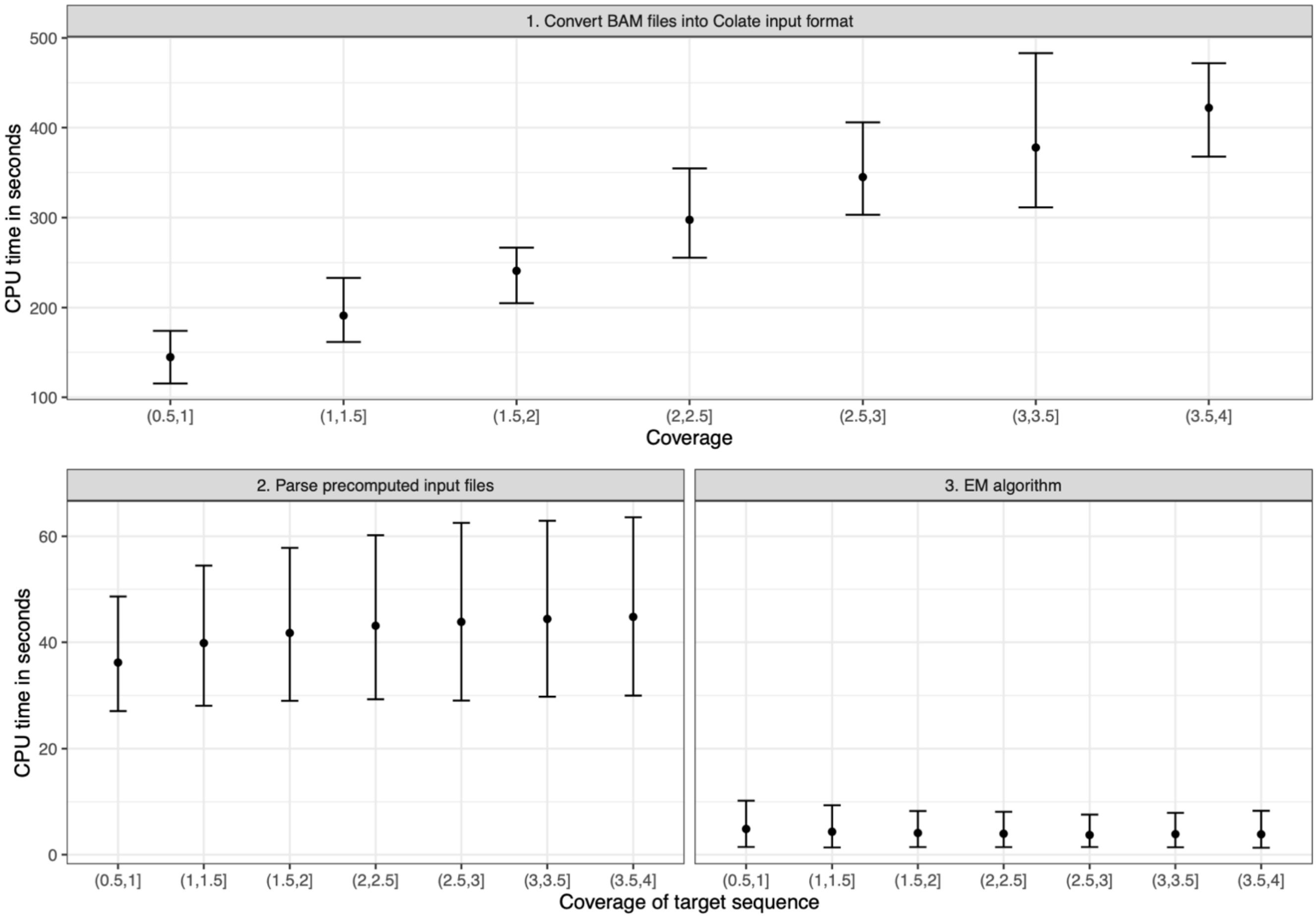

Supplementary Figure 1

Runtime of Colate on ancient genomes of <4x coverage, using mutations dated in a genealogy estimated using SGDP individuals (**Methods**). Step 1 converts BAM files into an input file format used for Colate, storing the number of reads supporting each allele at sites dated in the genealogy. This step is linear in coverage. Step 2 parses two sequences that were each processed using Step 1 and scales linearly with the number of mutations used in the analysis (which scales somewhat with increasing coverage as more mutations are included in the analysis). Step 3 infers maximum likelihood coalescence rates using an EM algorithm that is now independent of input sequence coverage and the number of mutations used in the analysis. The x-axis in steps 2 and 3 denotes the coverage of the target sequence; coverage of the reference sequence ranges from 0.5x to 4x and is reflected in the error bars. These runtimes were obtained on Intel E5-2640 Haswell CPUs running at 2.6GHz.

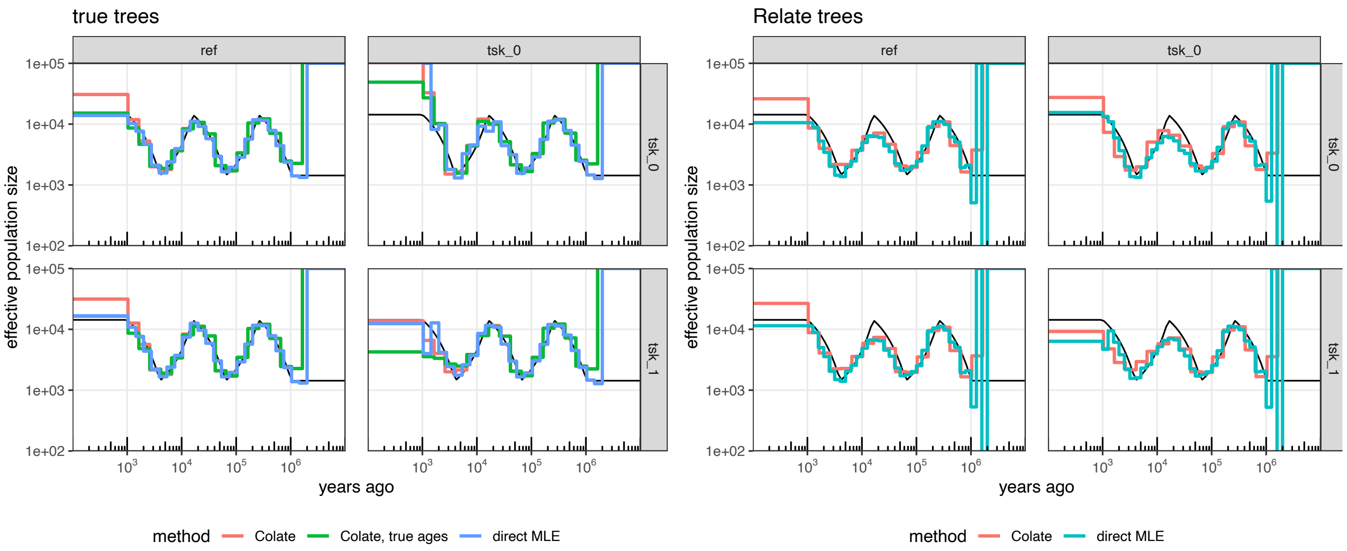

Supplementary Figure 2

Inferred effective population sizes using (**a**) true trees and (**b**) Relate trees for a stdpopsim simulation equivalent to whole human genomes of 102 diploid sequences with a sawtooth history and a human-like recombination map. We divide samples into a group of 100 diploid samples (ref), and two groups with one diploid sample each (tsk_0 and tsk_1). Rows show the target sequence used (tsk_0 or tsk_1) and columns show the reference sequences used (ref or tsk_0), where panel tsk_0 vs tsk_0 corresponds to the within individual effective population size. For the direct MLE, we use joint trees of all 102 samples. For Colate, we use trees corresponding to the 100 diploid samples (ref) to date mutations. We also evaluate Colate in the case where true mutation ages are known.

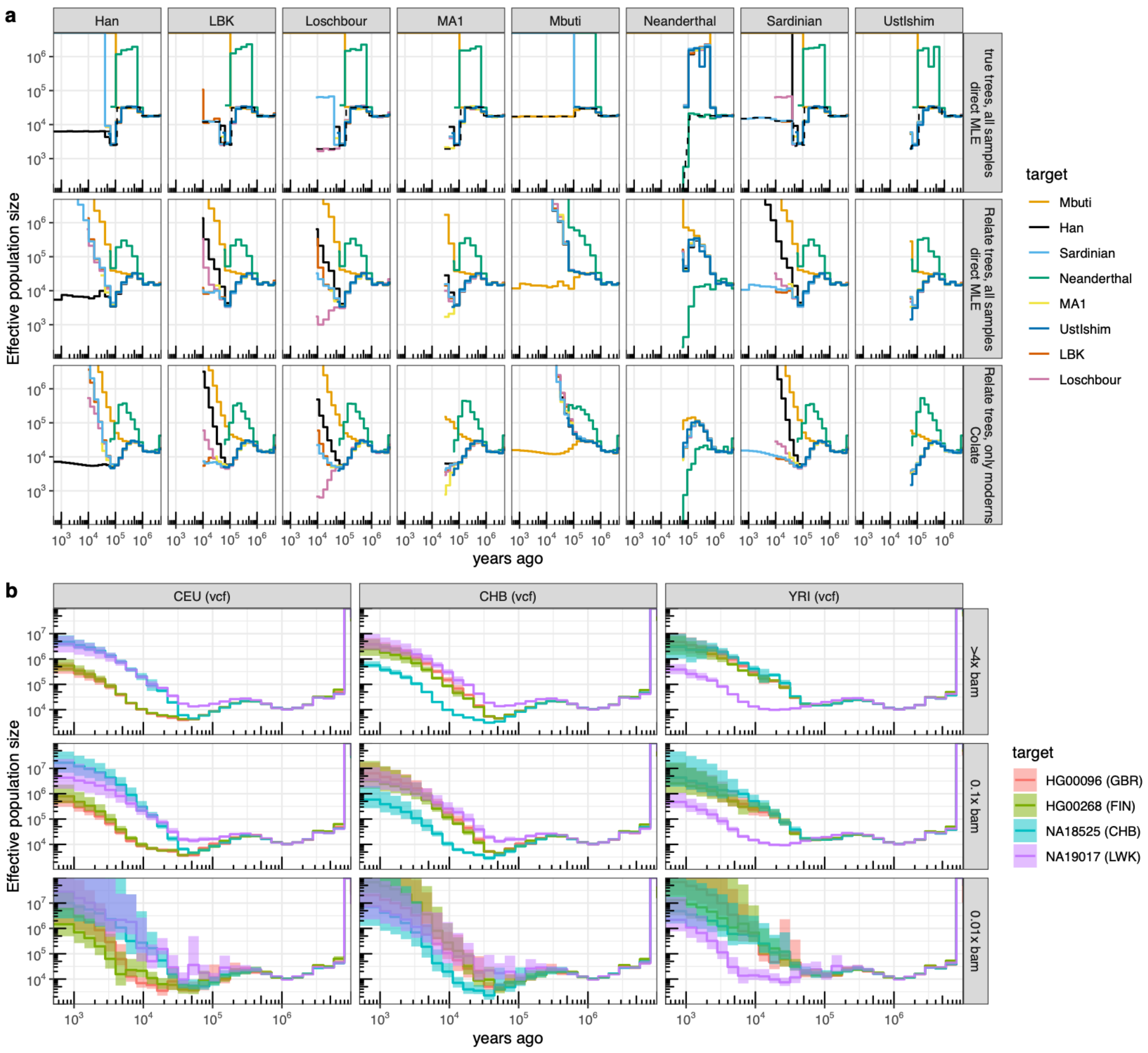

Supplementary Figure 3

a, Simulation of the multipopulation model of ancient Eurasia implemented in the stdpopsim package(Adrion et al. 2020; Kamm et al. 2020), also used in Figure 2**a**. This simulation includes three modern human groups (Mbuti, Han, and Sardinian) with 100 diploid sequences each, and five diploid ancient genomes. Coalescence rates were calculated using true genealogical (true trees; direct MLE), *Relate* trees of all samples (*Relate* trees; direct MLE), as well as *Colate*, where the genealogy for dating mutations included all modern human groups but not the ancients. Dashed line shows the true within-group population sizes in simulation. **b,** *Colate*-inferred coalescence rates between four 1000 Genomes Project samples (HG0096, HG00268, NA18525, NA19017) and the remaining 1000 Genomes samples in CHB, CEU, and YRI. The target samples are downsampled to 4x, 0.1x, and 0.01x mean coverage (rows). Confidence intervals are constructed using 100 block bootstrap iterations with a block size of 20Mb.

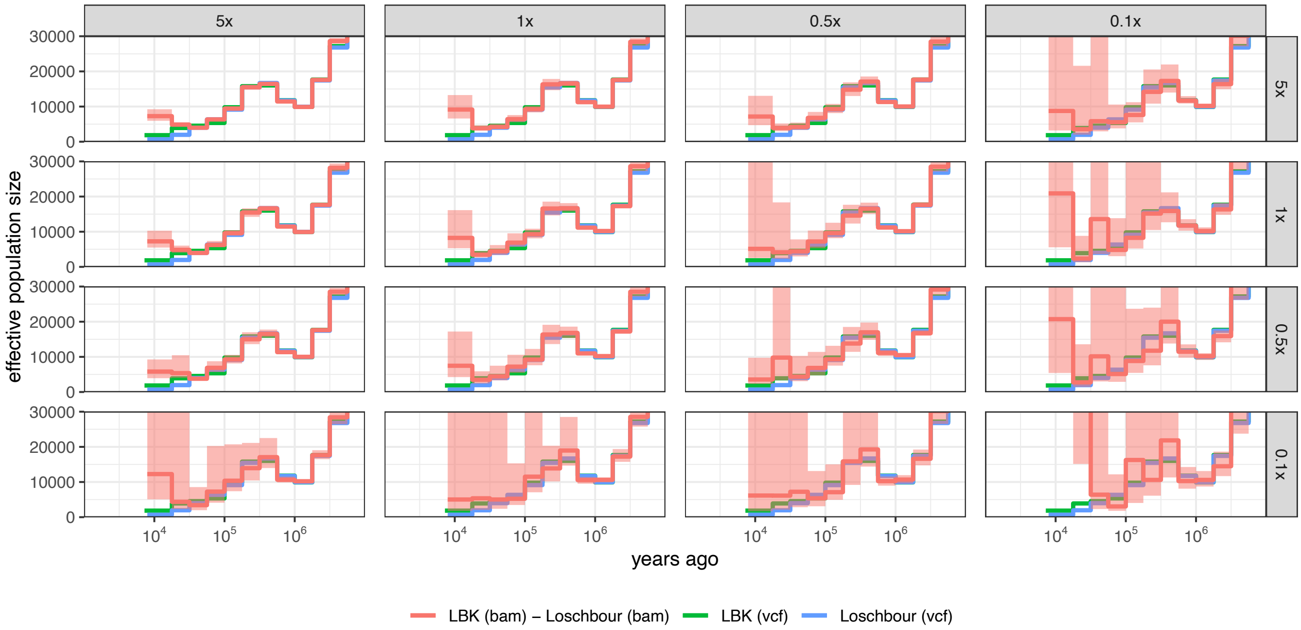

Supplementary Figure 4

Colate-inferred effective population sizes between LBK (target sample; rows) and Loschbour (reference sample; columns), with each individual downsampled to 5x, 1x, 0.5x, and 0.1x. We additionally also show the within individual effective population sizes for each individual in green and blue, which are identical in all panels and is calculated using VCFs that were called on the original BAM files (>10x coverage).

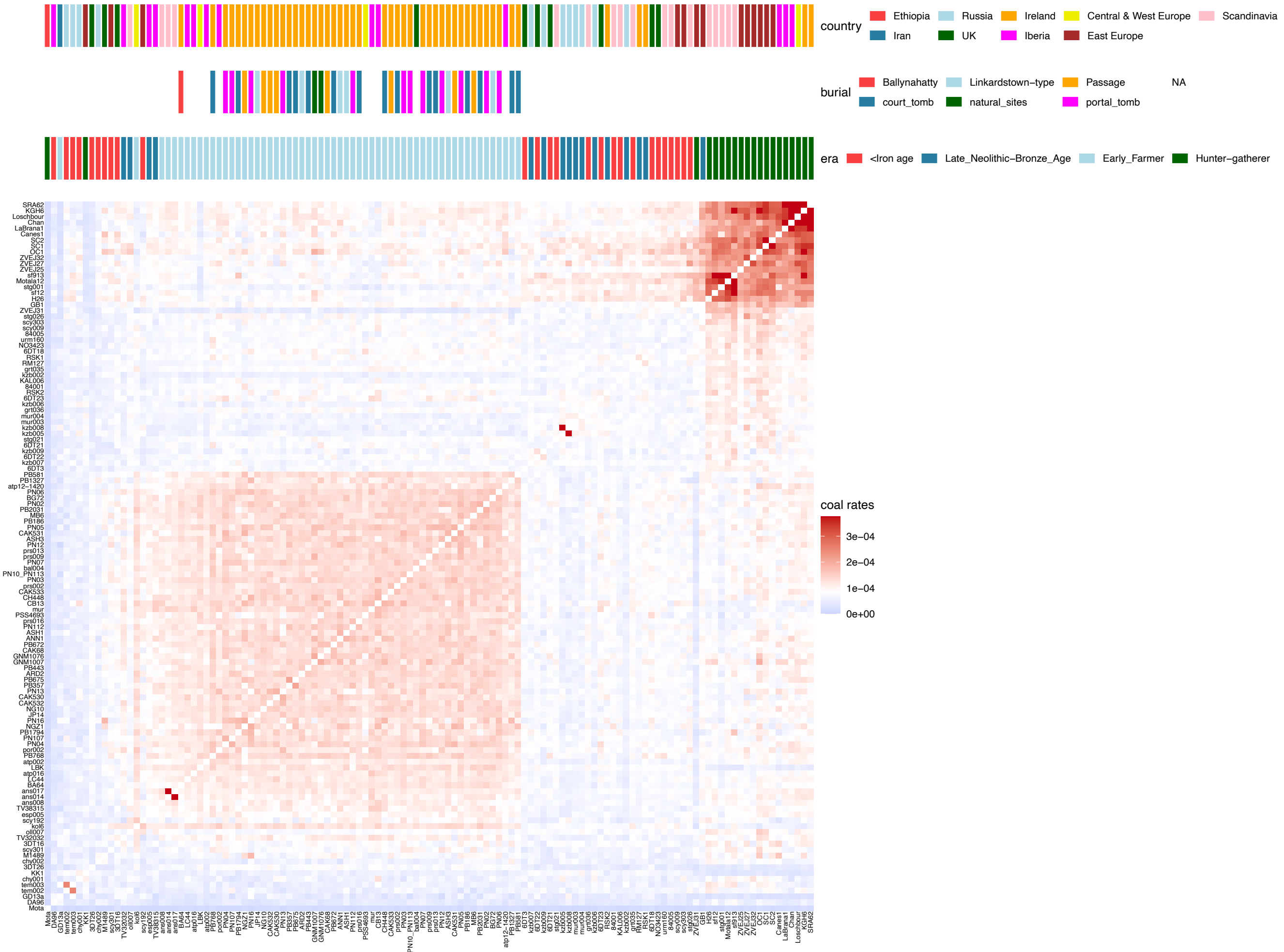

Supplementary Figure 5

Matrix of coalescence rates inferred using Colate for the epoch spanning from the age of the older sample of each row/column pair to 15,000 YBP. Rows and columns of the matrix are sorted by applying UPGMA. Annotations given at the top correspond to the geographical region of each sample, burial type for the Irish genomes, and time period.

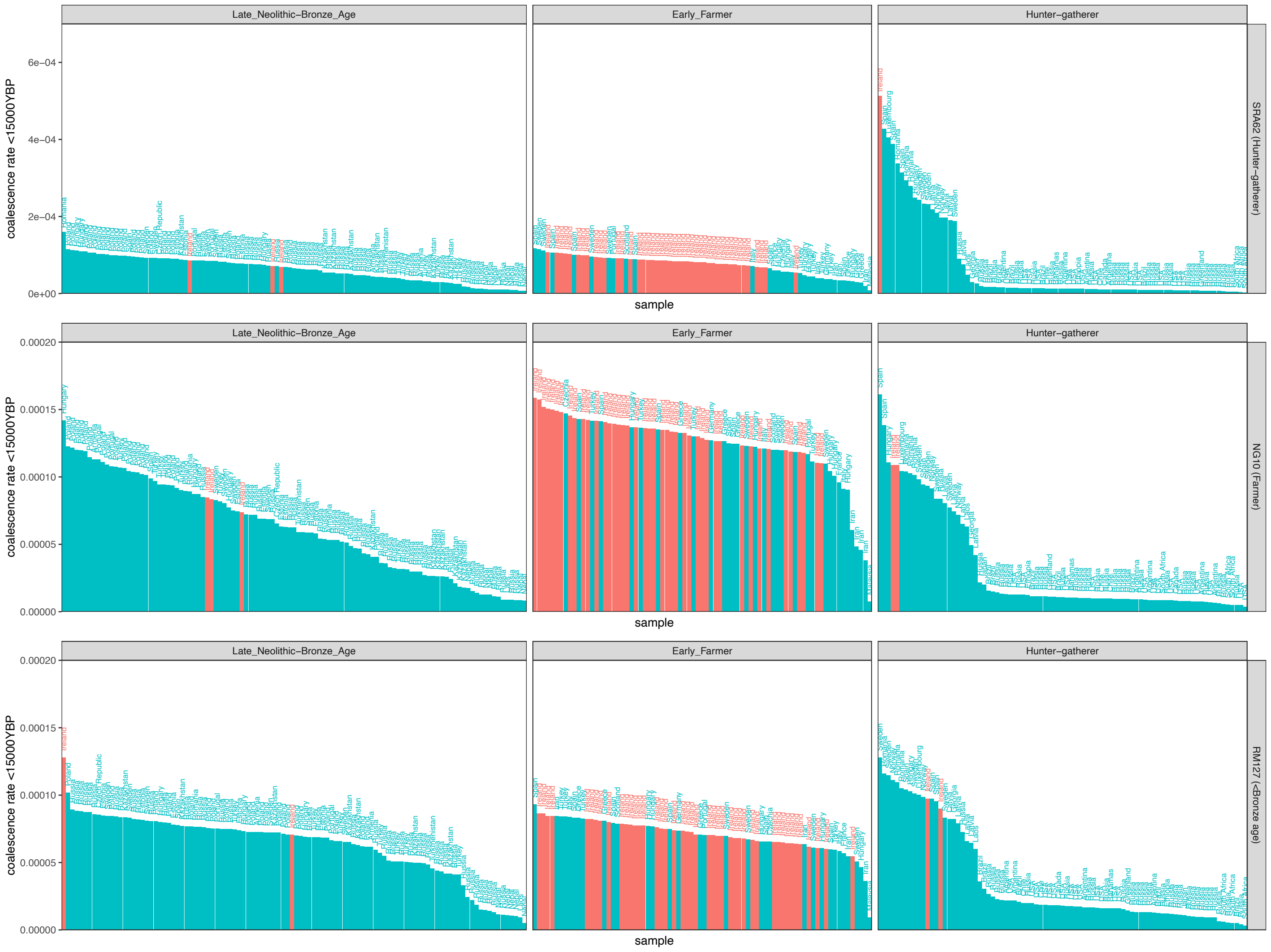

Supplementary Figure 6

Colate-estimated coalescence rate of an Irish HG (SRA62), Irish Neolithic farmer (NG10), and an Irish Bronze-age sample (RM127) to other ancient samples, calculated for an epoch ranging from the date of the sample to 15,000 years BP. In each panel, samples are sorted in descending order. Colours indicate Irish samples (red) and labels annotate geographic region.

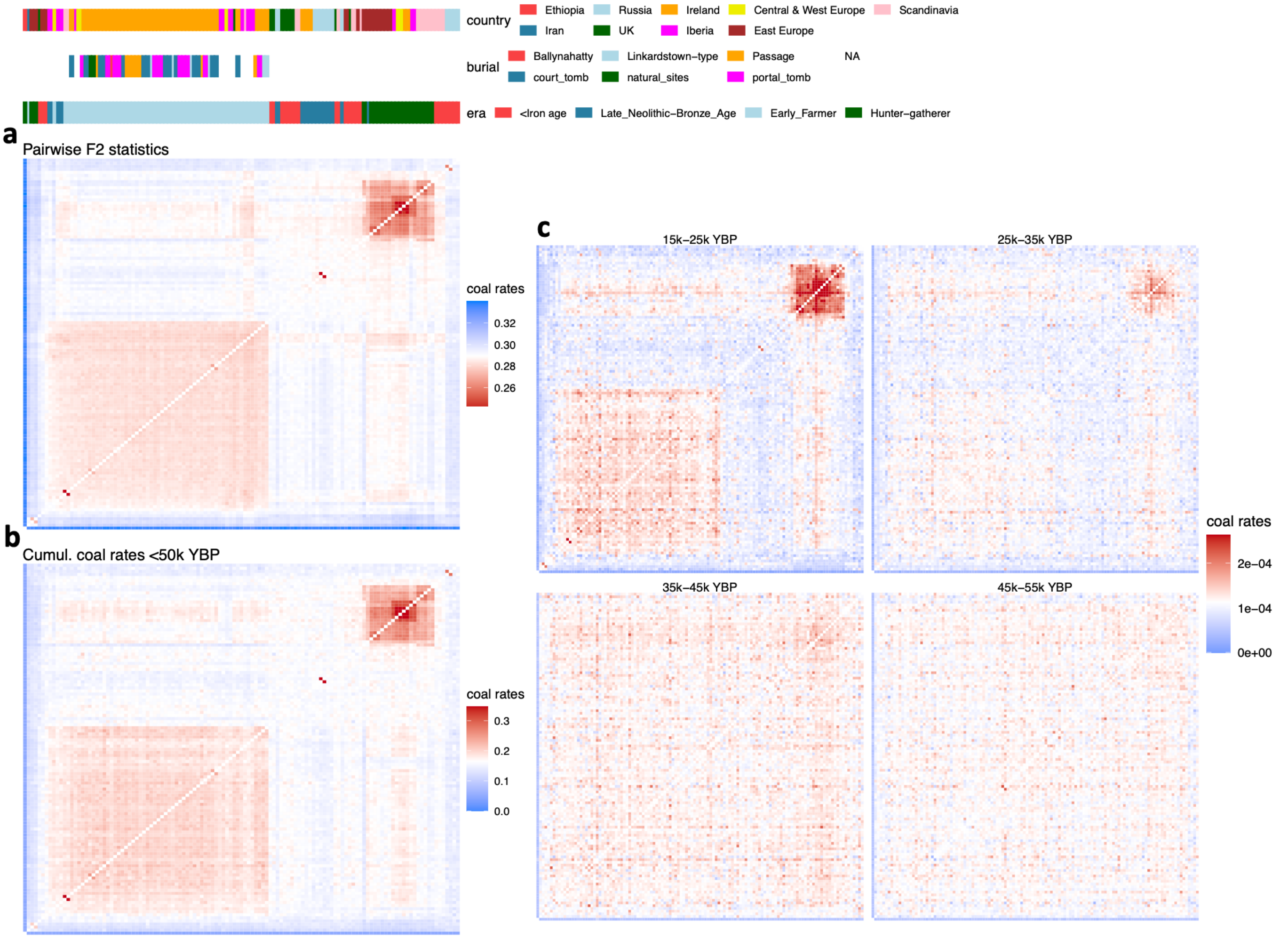

Supplementary Figure 7

a, Matrix containing pairwise F2 statistics calculated using pseudohaploid calls for each individual (**Methods**). Matrix is sorted by applying UPGMA. Annotations at the top correspond to geographical region of each sample, burial type for the Irish genomes, and time period. **b,** Matrix of Colate-inferred coalescence rates integrated over 0 – 50k YBP, ordered in the same way as the matrix in **a**. **c,** Matrices of pairwise coalescence rates for four additional epochs. All matrices are sorted in the same way as the matrix in **a**.

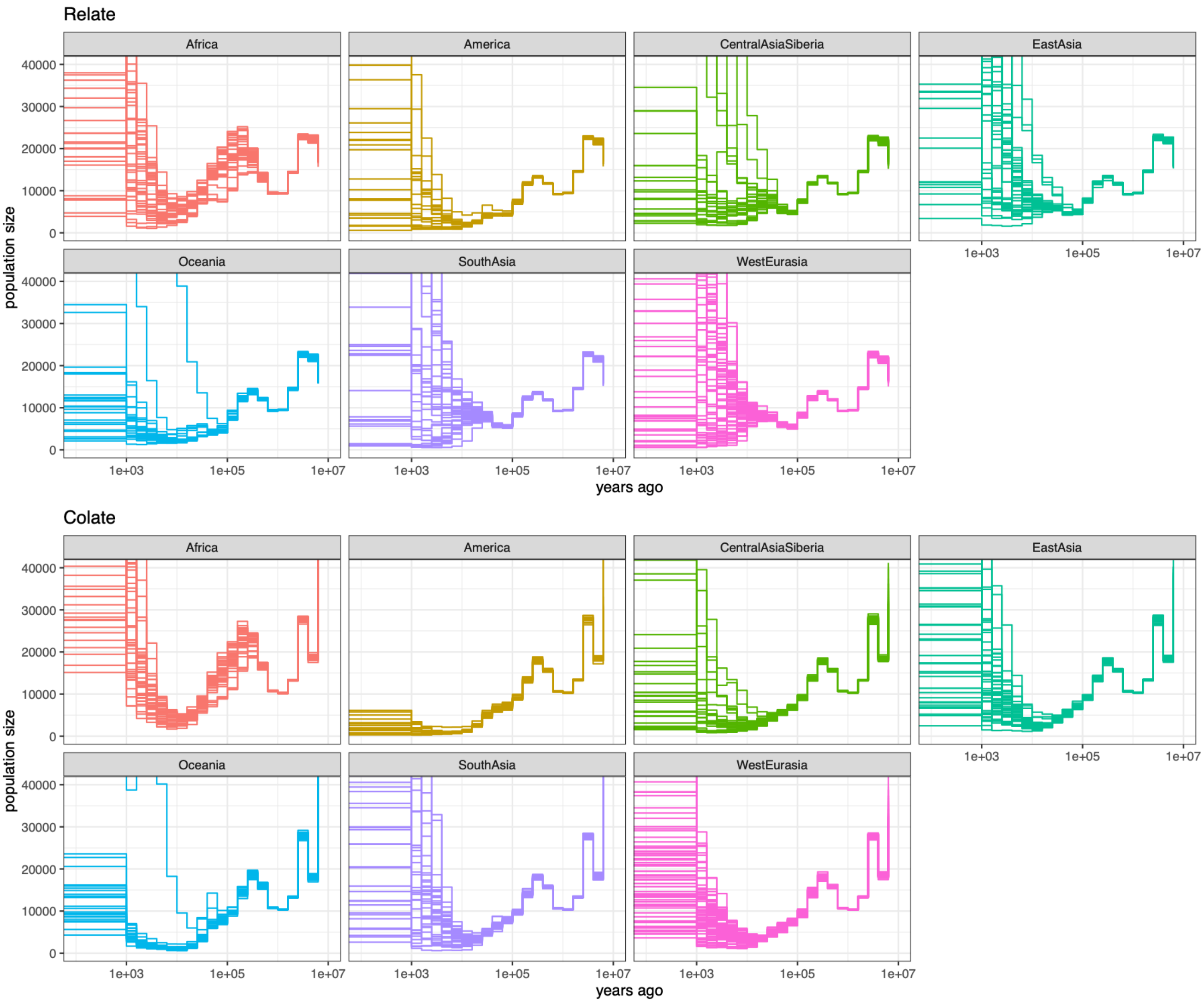

Supplementary Figure 8

Within-individual effective population sizes for 278 samples in the Simons Genome Diversity Project, estimated using *Relate* (top) and *Colate* (bottom).

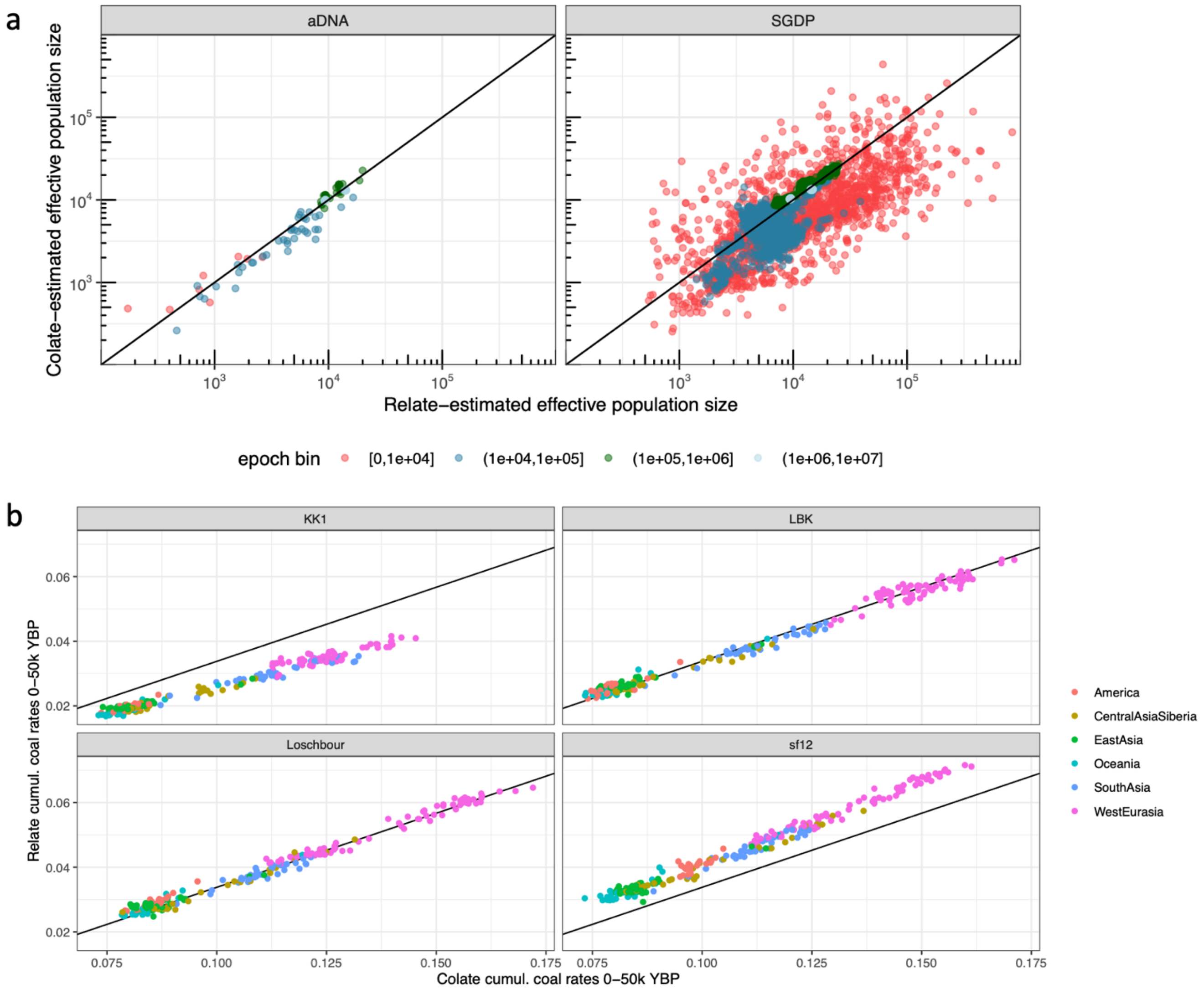

Supplementary Figure 9

a, *Colate*-estimated within individual effective population sizes plotted against their *Relate*-estimated equivalents. Epochs are grouped into four bins, shown by different colours. **b,** Coalescence rates between sample shown in facet title against non-African SGDP individuals, integrated over 0 – 50k YBP, compared between *Relate* and *Colate*. We performed a linear regression on all four samples jointly, with the line shown corresponding to y = 0.46x-0.01, which was used to rescale *Colate* coalescence rates in Figure 4c.

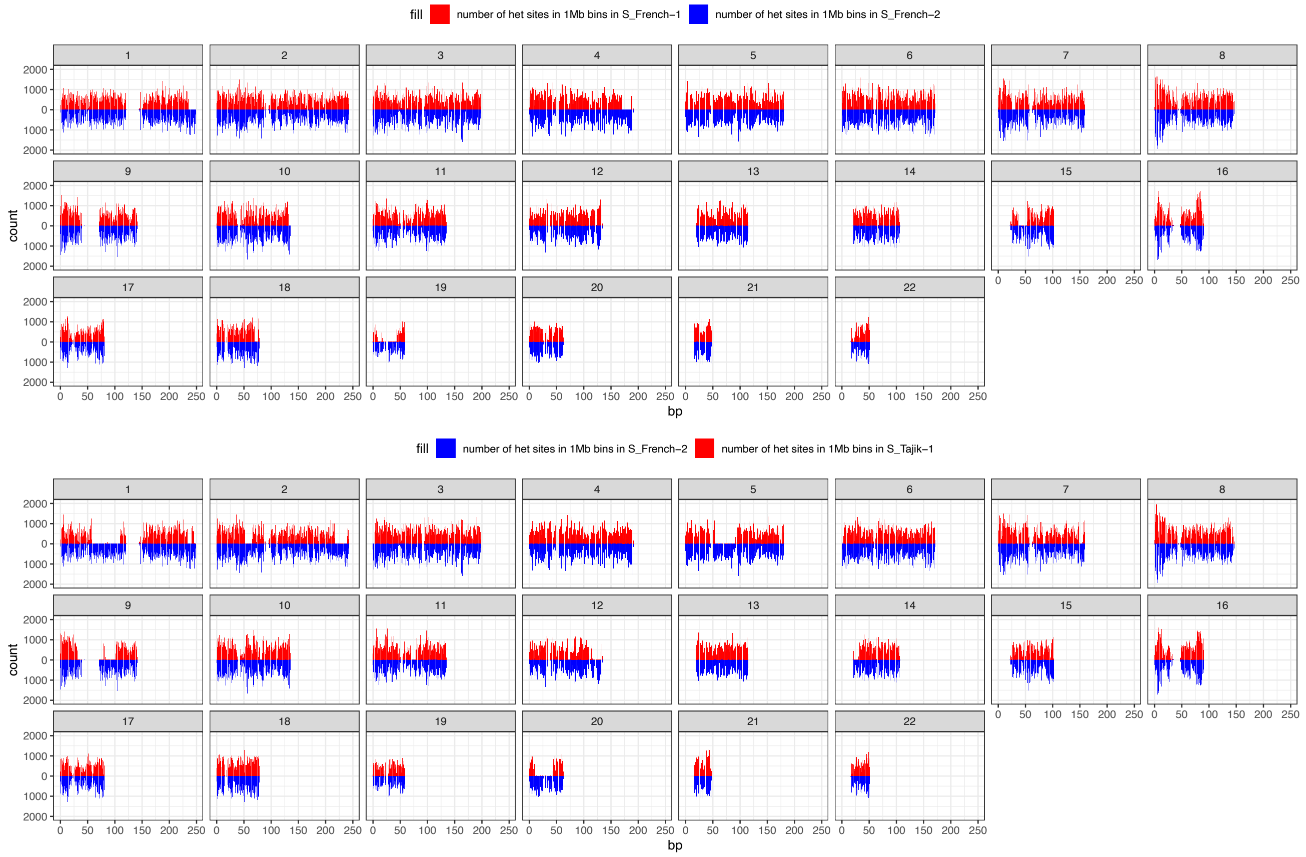

Supplementary Figure 10

Number of heterozygous sites in 1Mb bins for the SGDP samples S_French-1 and S-Tajik-1 (red in top and bottom plot) compared to S_French-2 (blue in both plots), showing long runs of homozygosity (ROH) in S_French-1 and S_Tajik-1 compared to S_French-2. These ROH appear in different locations in S_French-1 and S_Tajik-1. While S_French-1 is a cell line, which could artificially introduce such ROH, S_Tajik-1 is a blood sample. The *Relate*-inferred effective population sizes in the most recent bin for these individuals are 10,898 for S_French-1, 161,112 for S_French-2, and 909 for S_Tajik-1.

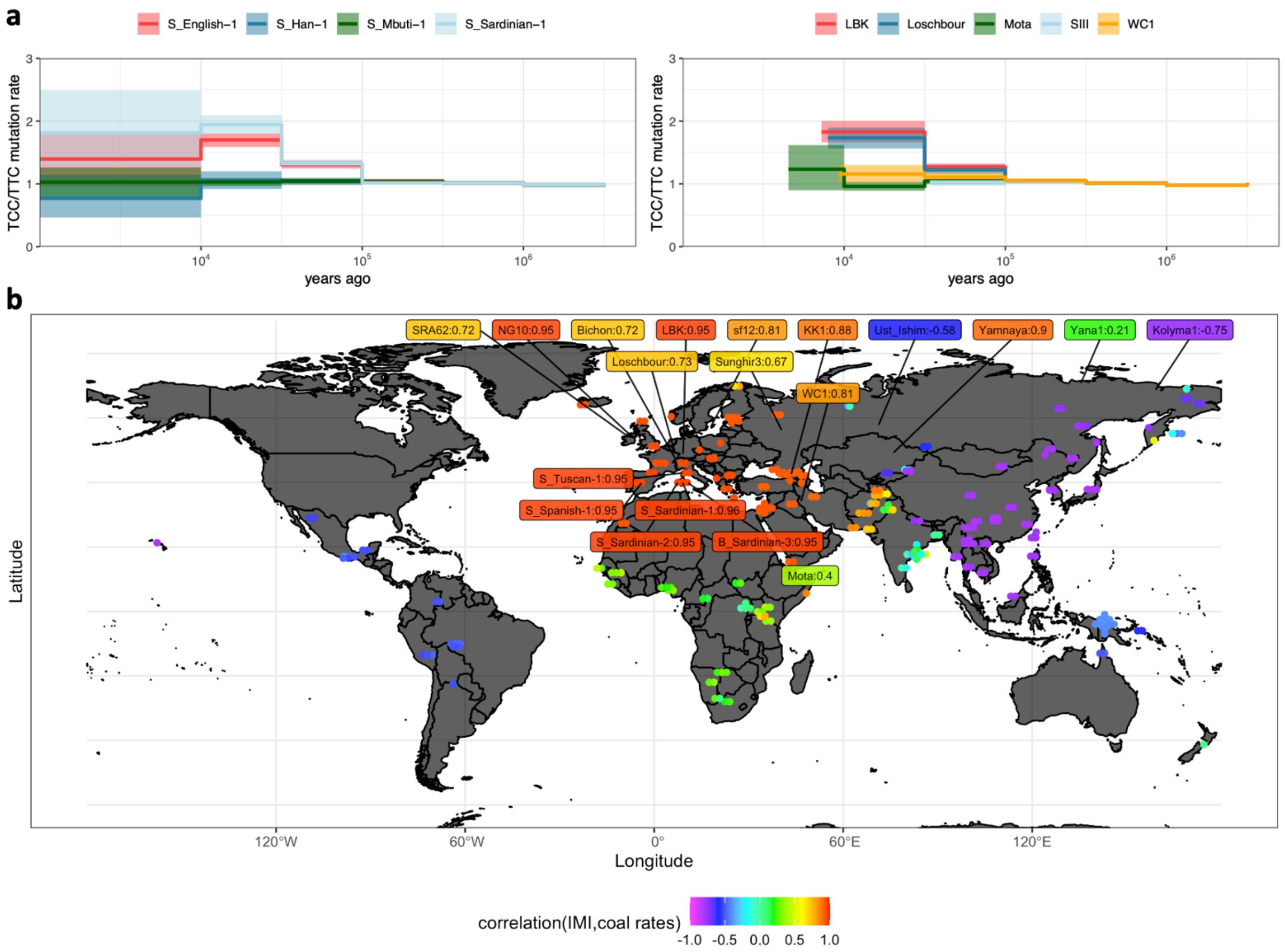

Supplementary Figure 11

a, TCC/TTC mutation rate relative to the mutation rate in the time interval 100k-1M YBP for four modern individuals and five ancient individuals. **b**, Correlation calculated between the "integrated mutation intensity" (IMI) of the TCC/TTC mutation rate (**Methods**) and Colate-inferred coalescence rates to all non-African SGDP individuals and non-Africans ancient samples. Correlations for SGDP individuals are shown by circles and correlations for ancient individuals, as well as the top five modern samples, are labelled.

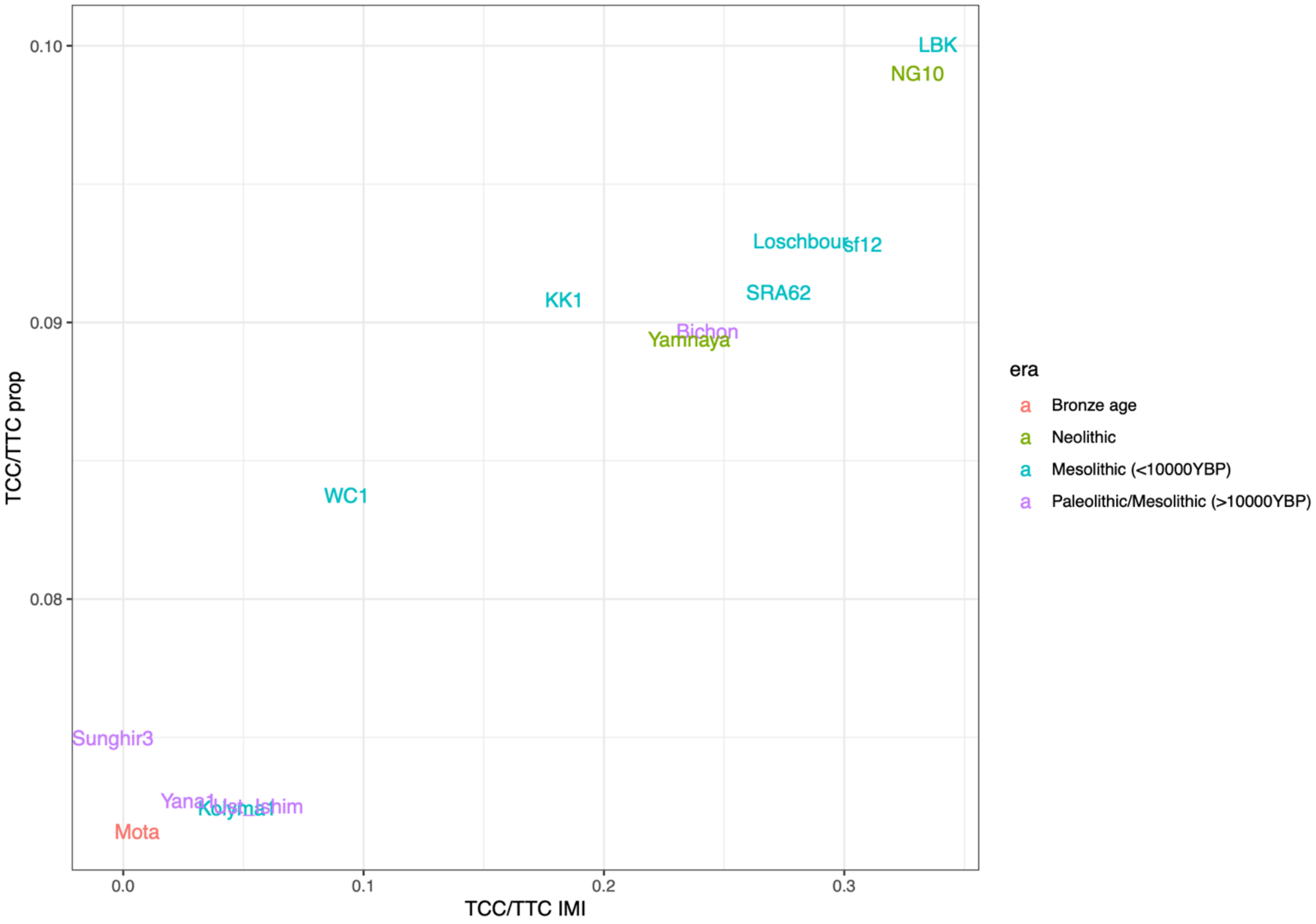

Supplementary Figure 12

Comparison of two different ways of quantifying the TCC/TTC mutation rate signature plotted against each other (**Methods**). X-axis shows the integrated mutation intensity (IMI) calculated from mutation rates directly obtained using *Relate* genealogies, whereas y-axis shows the number of TCC/TTC mutations relative to other transitions (excl. CpGs), for mutations dated to be younger than 100k YBP.

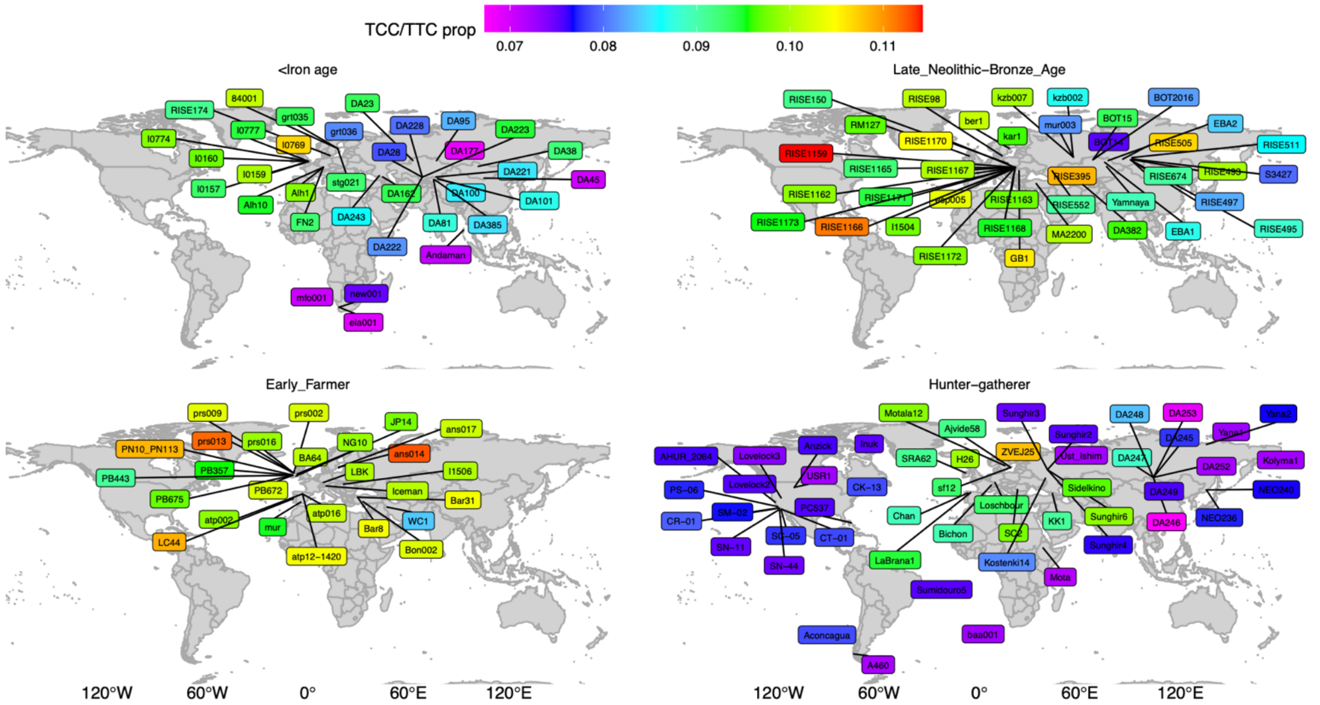

Supplementary Figure 13

The proportion of TCC/TTC mutations relative to C/T transitions (excluding those in CpG contexts) (**Methods**), for ancient samples of >2x mean coverage. Each map shows a different cultural context/time period and colours indicate signal strength.

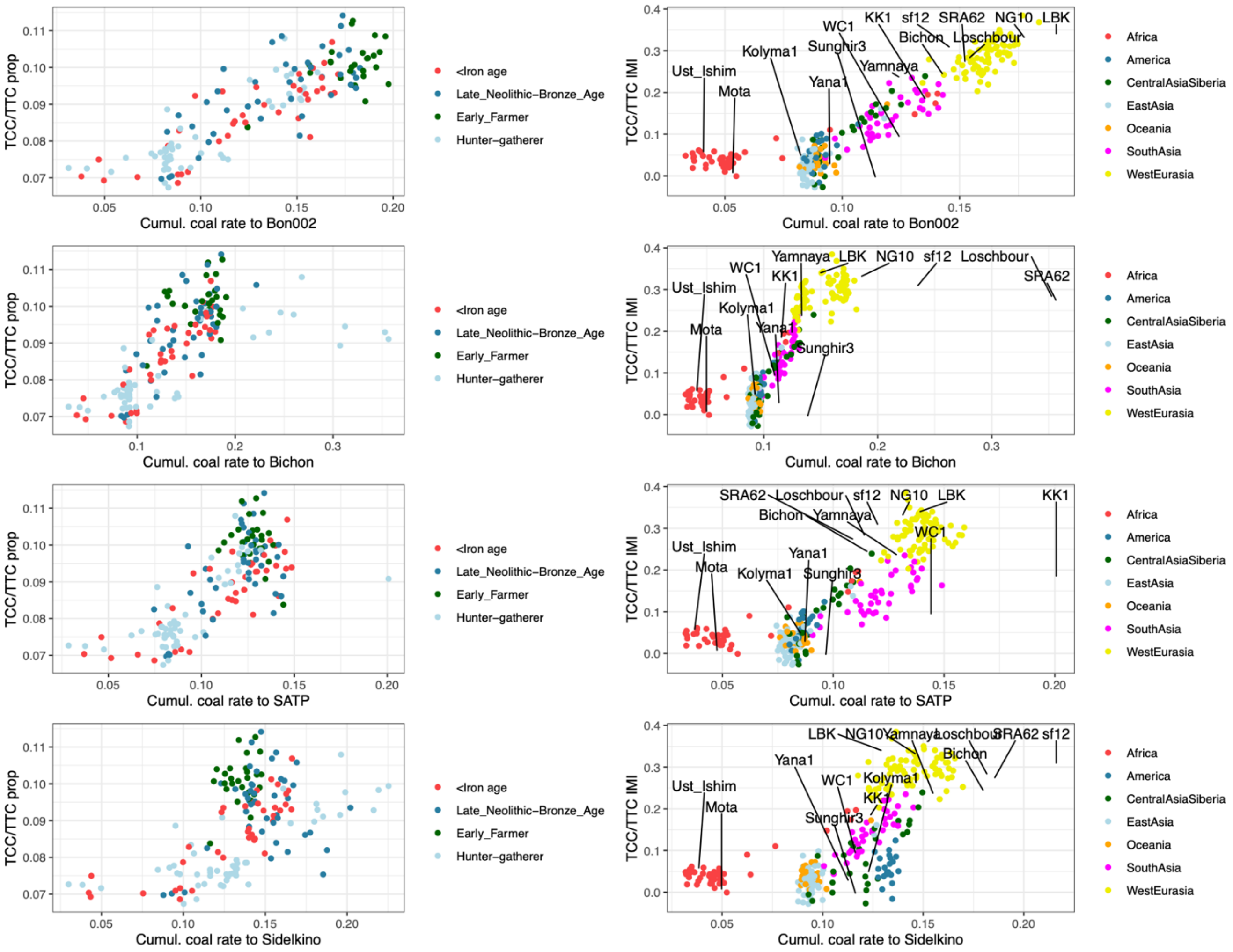

Supplementary Figure 14

Strength of the TCC/TTC mutation rate signal, quantified using the proportion of TCC/TTC mutations relative to transitions (left column) or area under the mutation rate curve (right column) (**Methods**) plotted against cumulative coalescence rates with Bon002, a 10k-year-old Anatolian individual, Bichon, a 13k-year-old Western HG, SATP, a 13k-year-old Caucasus HG, and Sidelkino, a 11k-year-old Eastern HG. The cumulative coalescence rates are calculated as the integral of the coalescence rate from sample age to 50k YBP.

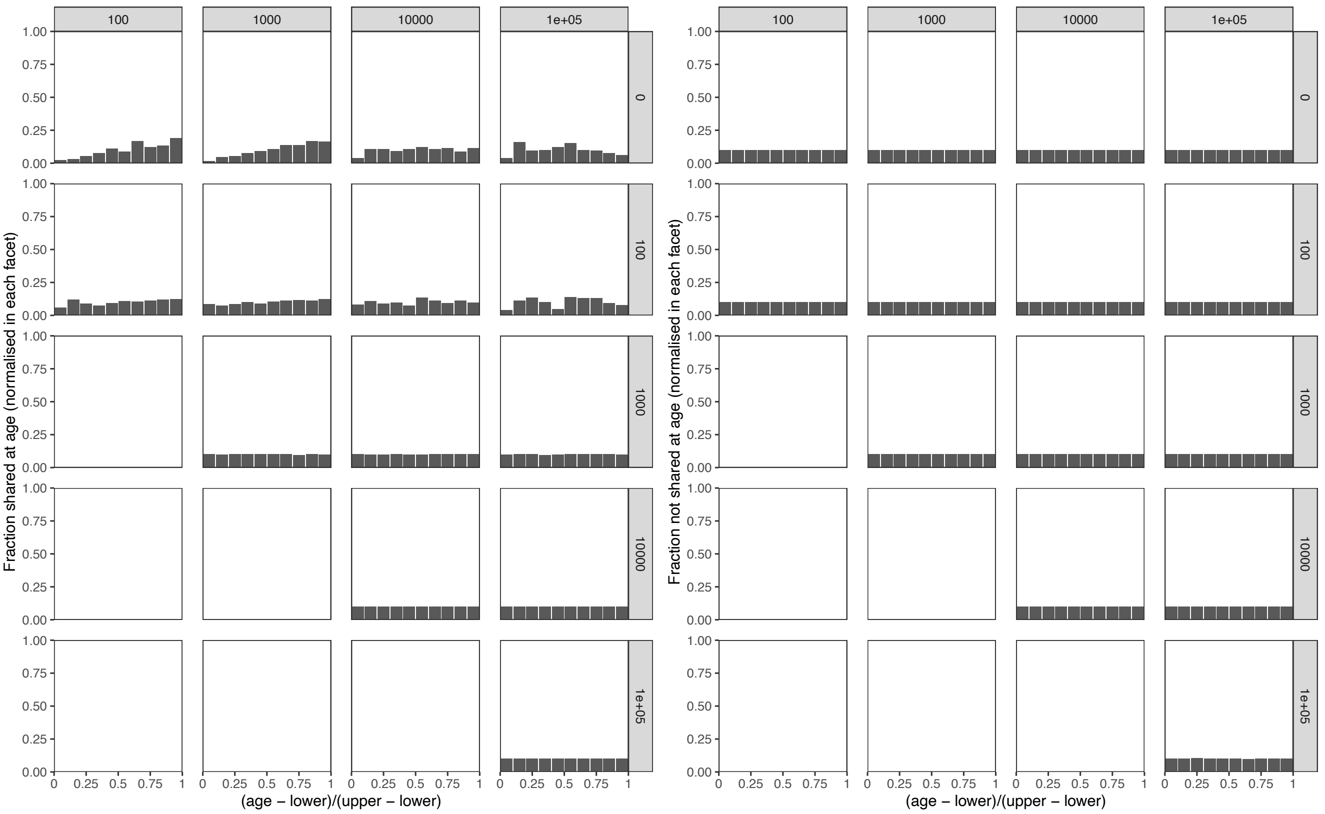

Supplementary Figure 15

For mutations segregating in the 100 diploid samples (ref) in the zigzag simulation of Supplementary Figure 2, we plot a histogram of the true age of the mutation relative to lower and upper ages of the coalescence events of the branch on which this mutation occurred, using the genealogy of these 100 diploid samples only and stratified by whether or not it is shared with sample tsk_0 (left and right panel). We additionally stratify by age bins of lower (rows) and upper (columns) coalescence ages. This shows that mutations that are singletons in the group of 100 diploid samples and are shared with tsk_0 have a non-uniform age, whereas all other categories possess age distributions close or nearly identical to uniform distributions.
